# Unexpected White Phosphorus (P_4_) Activation Modes with Silylene‐Substituted *o*‐Carboranes and Access to an Isolable 1,3‐Diphospha‐2,4‐disilabutadiene

**DOI:** 10.1002/anie.202205358

**Published:** 2022-05-19

**Authors:** Yun Xiong, Shicheng Dong, Shenglai Yao, Jun Zhu, Matthias Driess

**Affiliations:** ^1^ Department of Chemistry: Metalorganics and Inorganic Materials Technische Universität Berlin Strasse des 17. Juni 135, Sekr. C2 10623 Berlin Germany; ^2^ State Key Laboratory of Physical Chemistry of Solid Surface and Collaborative Innovation Center of Chemistry for Energy Materials (iChEM) College of Chemistry and Chemical Engineering Xiamen University Xiamen 361005 P. R. China

**Keywords:** Carboranes, Phosphasilenes, Phosphorus, Silicon, Small Molecule Activation

## Abstract

New types of metal‐free white phosphorus (P_4_) activation are reported. While the phosphine‐silylene‐substituted dicarborane **1**, CB‐**SiP** (CB=*ortho*‐C,C′‐C_2_B_10_H_10_, **Si**=PhC(tBuN)_2_Si, **P**=P[N(tBu)CH_2_]_2_), activates white phosphorus in a 2 : 1 molar ratio to yield the P_5_‐chain containing species **2**, the analogous bis(silylene)‐substituted compound **3**, CB‐**Si_2_**_,_ reacts with P_4_ in the molar ratio of 2 : 1 to furnish the first isolable 1,3‐diphospha‐2,4‐disilabutadiene (Si=P−Si=P‐containing) compound **4**. For the latter reaction, two intermediates having a CB‐Si_2_P_4_ and CB‐Si_2_P_2_ core could be observed by multinuclear NMR spectroscopy. The compounds **2** and **4** were characterized including single‐crystal X‐ray diffraction analyses. Their electronic structures and mechanisms were investigated by density functional theory calculations.

## Graphical abstract

New metal‐free white phosphorus (P_4_) activation modes have been observed using divalent Si^II^ atoms bonded to a 1,2‐dicarborane scaffold: while two molar equivalents of the silylene‐phosphine **1** are capable of co‐activating one molecule of white phosphorus, yielding the P_5_‐chain containing species **2**, the degradation of P_4_ using the bis(silylene) analogue **3** affords the first isolable 1,3‐diphospha‐2,4‐disilabutadiene **4** with a Si=P−Si=P delocalized skeleton.

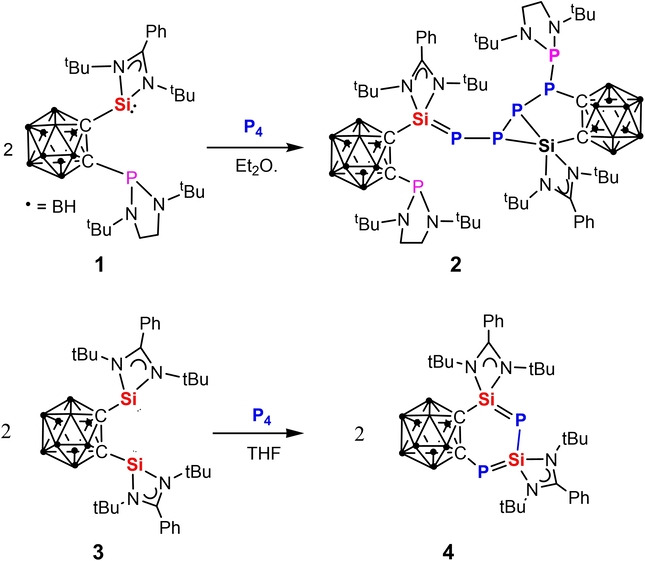

Degradation and direct functionalization of white phosphorus (P_4_) into molecular phosphorus compounds is of fundamental importance in academia and industry.^[1]^ In comparison with the extensively investigated chlorine‐free functionalization of P_4_, mediated by transition‐metals,[^2^, ^3^] P_4_ activation assisted by main‐group elements in low oxidation states is less developed.[^4^, ^5^, ^6^] Recent reports demonstrated that low‐valent Group 14 species, such as cyclic (alkyl)(amino)carbenes (cAACs), *N*‐heterocyclic carbenes (NHCs) and *N*‐heterocyclic silylenes (NHSis), represent suitable building blocks to access functional phosphorus compounds with diverse structural motifs.^[6]^ For instance, via degradation or aggradation of P_4_ with NHCs and cAACs, several carbene‐functionalized P_*n*_ (*n*=1, 2, 4, 8, 12) adducts have been realized,[^7^, ^8^, ^9^, ^10^] among which **A** has been trapped as a reaction intermediate by using 2,3‐dimethyl‐1,3‐butadiene (Figure 1).^[10]^ On the other hand, di‐coordinate silylenes,^[11]^ including a transient silylene, activate P_4_ through insertion of the divalent Si atom into one or two P−P bonds to furnish silyl‐functionalized phosphorus compounds featuring Si_*n*_P_4_ cages (*n*=1 and 2),[^12^, ^13^, ^14^, ^15^] including **B**. The latter are reminiscence of P_4_ activation products using disilenes as starting materials, yielding butterfly‐like Si_2_P_2_ heterobicyclo[1.1.0]butanes via insertion of Si^II^ atoms into P−P bonds.^[16]^ Notably, more electron‐rich mono‐ and bis‐silylenes with three coordinate Si^II^ atoms are capable to activate P_4_ in a similar fashion as carbenes, yielding products featuring Si=P bonds (**C** in Figure 1).^[14]^ Likewise, compound **D** resulted from P_4_ activation with the hypercoordinated disilylene LSi−SiL [L=PhC(N*t*Bu)_2_].^[17]^

**Figure 1:**
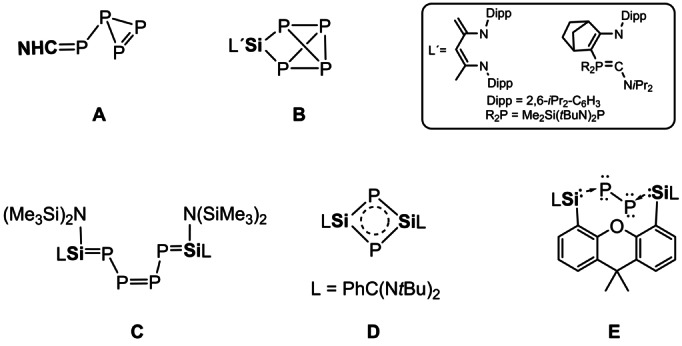
Reaction intermediate **A** and the isolable species **B**–**E** resulting from P_4_ activation with NHCs, cAACs, NHSis, disilylene and bis(silylene).

Quite recently, starting from a 9,9‐dimethyl‐4,5‐xanthenediyl bis(silylene),^[18]^ its reaction with P_4_ resulted in the formation of the isolable bis(silylene)‐supported P_2_ complex **E** which acts as an anionic monophosphorus transfer reagent.^[19]^ This result prompted us to examine P_4_ activation utilizing related chelating mono‐ and bis‐silylenes with different ligand scaffolds. Employing the recently reported *o*‐dicarborandiyl‐supported phosphine‐silylene CB‐**SiP 1** (CB=*ortho*‐C,C′‐C_2_B_10_H_10_; **Si**=PhC(*t*BuN)_2_Si; **P**=P[N(*t*Bu)CH_2_]_2_)^[20]^ and the corresponding bis(silylene) CB‐**Si_2_ 3**,^[21]^ we now learned that they show very different P_4_ activation modes. Herein we wish to report on these different activation modes of **1** and **3**.

Treatment of **1** with P_4_ in the molar ratio of 2 : 1 at room temperature in diethyl ether furnishes a yellow precipitate of **2** in 84 % isolated yields (Scheme 1). In fact, regardless of the molar ratio of the two reactants, **2** was observed as sole product. Akin to the formation of the NHC‐supported intermediate **A** (Figure 1),^[10]^ we propose the formation of the initial intermediate **1⋅P_4_** (Scheme 1), which reacts with a second molar equivalent of **1** via [1+2]‐cycloaddition to afford the intermediate **2**′. Presumably, owing to the steric congestion in **2′**, it isomerizes to **2** as the final product involving a P atom insertion reaction into the nearby C−P bond and formation of a new Si−P bond.

**Scheme 1:**
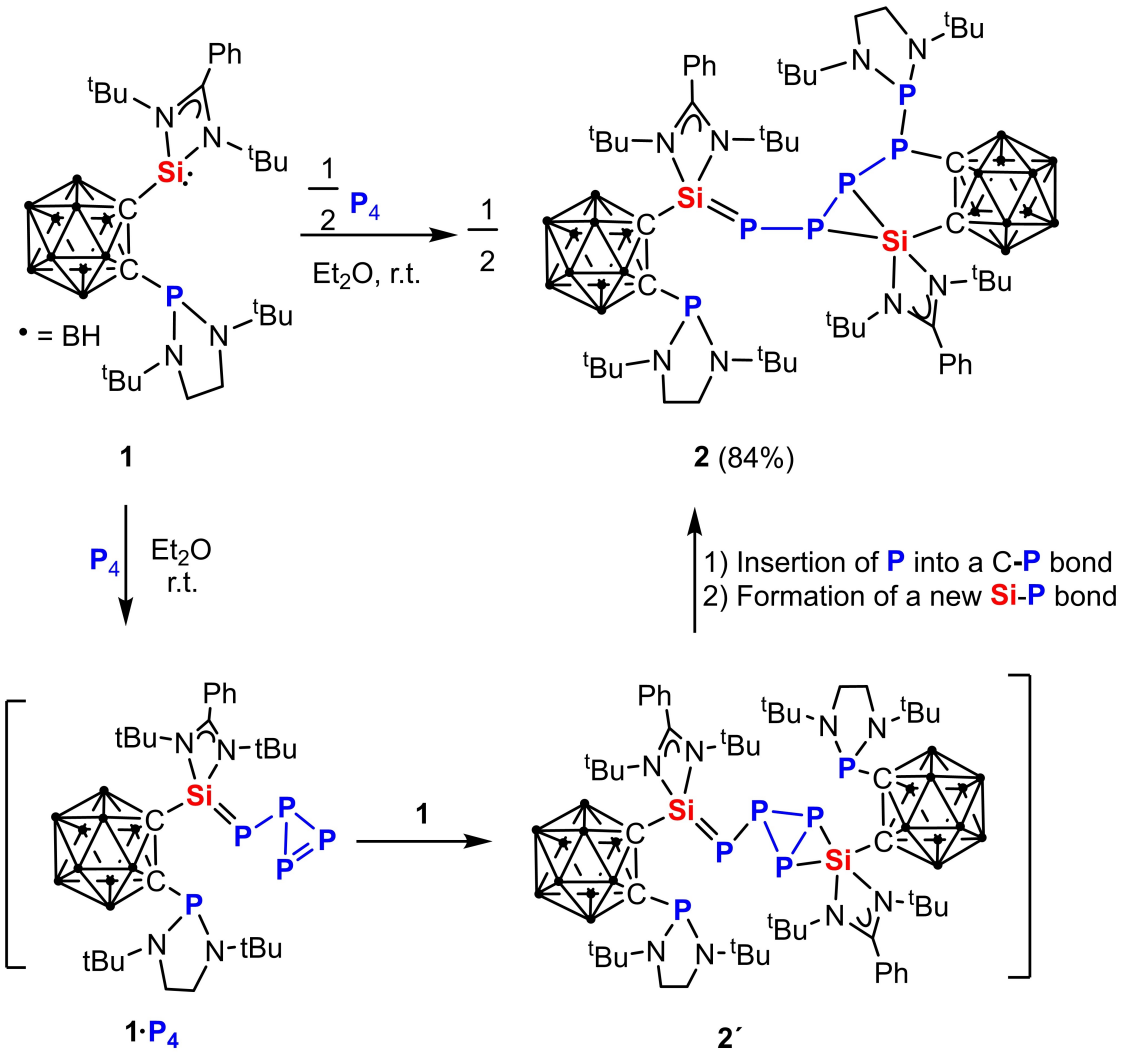
Reaction of **1** with P_4_ affording **2** via **1⋅P_4_** and **2′**, respectively.

As expected, four proton signals are observed for the four *t*Bu groups of the two different silylene moieties in **2** in the ^1^H‐NMR spectrum, while the four *t*Bu groups in the two P(N(*t*Bu)CH_2_)_2_ moieties give rise to only two ^1^H singlets. In the ^31^P{^1^H}‐NMR spectrum of **2**, the six chemically inequivalent ^31^P nuclei show six different multiplets (Figure 2). The assignment of the resonances for these P atoms is supported with density functional theory (DFT) calculations of the chemical shifts (Figure 2, Figure S15 in Supporting Information). The data show that all three sorts of P atoms bonded to the silicon atoms in **2** give signals in the high field region at *δ* (ppm)=−145.8 (*P4*), −191.8 (*P5*), and −225.5 (*P6*), respectively, due to the Si^δ+^−P^δ−^ bond polarity. In contrast, the three resonances at *δ*=135.5 (*P2*), 106.8 (*P1*), and 40.0 (*P3*) represent the resonances of the P atoms bonded either with electron‐withdrawing carborane cage C atoms or with a P atom of the P(N*t*BuCH_2_)_2_ moiety. It is noteworthy that the resonance of the unreacted di(amino)phosphine‐P atom (*δ*=106.8 ppm) is close to that in the precursor **1** (*δ*=102.9 ppm). Accordingly, in the ^29^Si{^1^H}‐NMR spectrum of **2**, the four‐coordinate Si1 atom resonates in low‐field region at *δ*=39.2 ppm, which is even down‐field shifted compared with that of the three‐coordinate Si atom in **1** (*δ*=16.8 ppm) and reflects the aforementioned polarity of the Si=P bond. In contrast, the five‐coordinate Si2 atom in **2** shows an up‐field signal at *δ*=−68.0 ppm.

**Figure 2:**
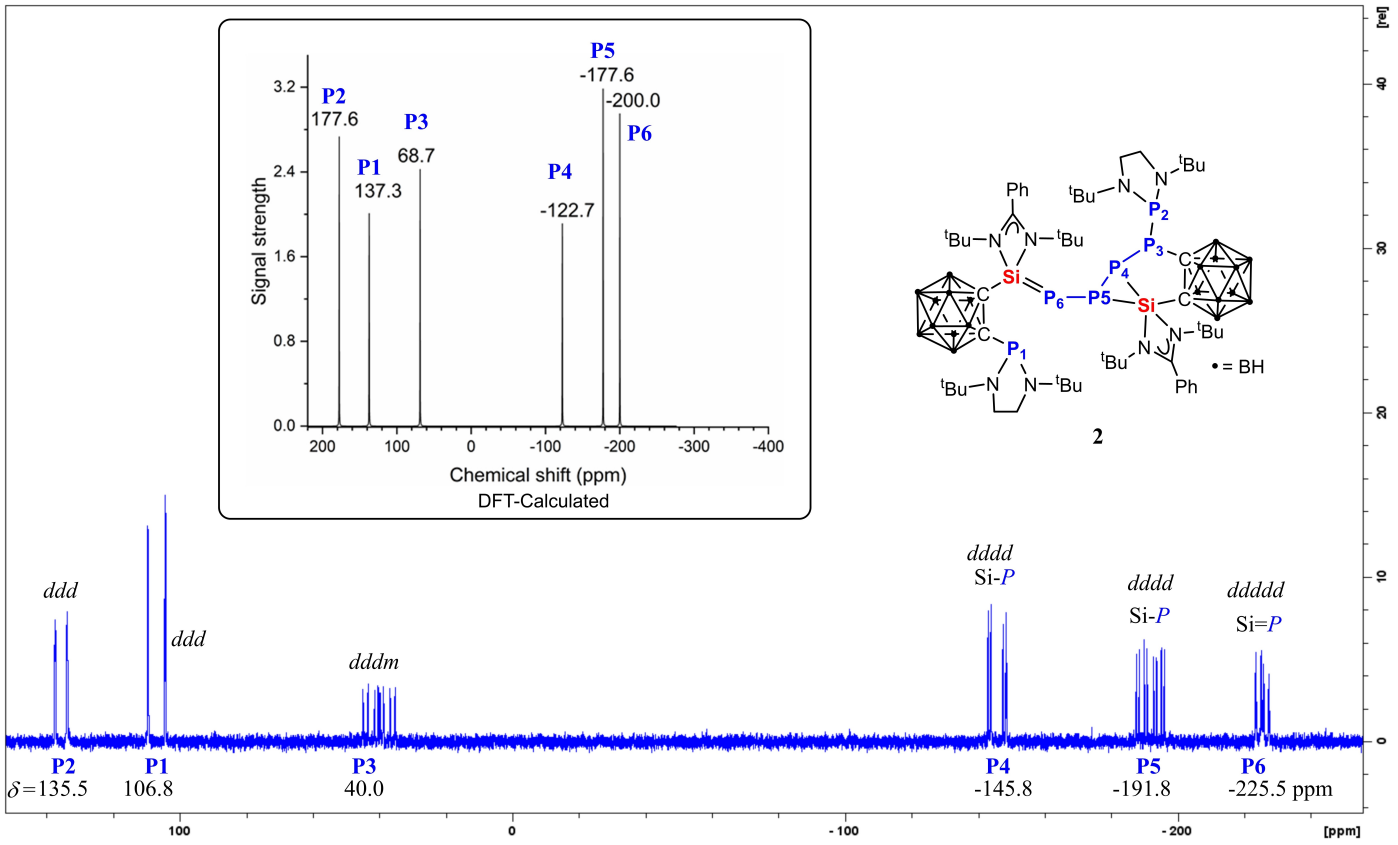
^31^P{^1^H}‐NMR spectrum of **2** in *d*_8_‐THF. Insert: DFT‐calculated chemical shifts for the respective ^31^P nuclei.

The molecular structure of **2** was established by a single‐crystal X‐ray diffraction analysis. It crystallizes in the triclinic space group *P*‐1 (Figure 3). **2** features a phosphane moiety and a phosphanyl‐containing P_5_ chain linked to two carborane cages and two silicon atoms. The P6 atom is two‐coordinate with a Si1−P6 bond of 2.132(1) Å, which is slightly shorter than the Si=P bonds in **C** (2.160(1) Å)^[14]^ and **D** (2.174(1) Å),^[17]^ close to that in **E** (2.130(1) Å.^[19]^ DFT calculations confirmed the latter Si1‐P6 bonding mode in **2** (Supporting Information, Figure S16). In comparison, the Si2−P4 and Si2−P5 distances (2.317(1) and 2.228(1) Å) in **2** are significantly longer than that of Si1=P6. While Si1 atom in **2** is four‐coordinate and adopts a distorted tetrahedral coordination geometry, the Si2 atom is bonded to two phosphorus atoms and has a distorted trigonal‐bipyramidal coordination geometry with the N8 and P4 atoms at apical positions (N8‐Si2‐P4 angle: 161.3(1)°).

**Figure 3:**
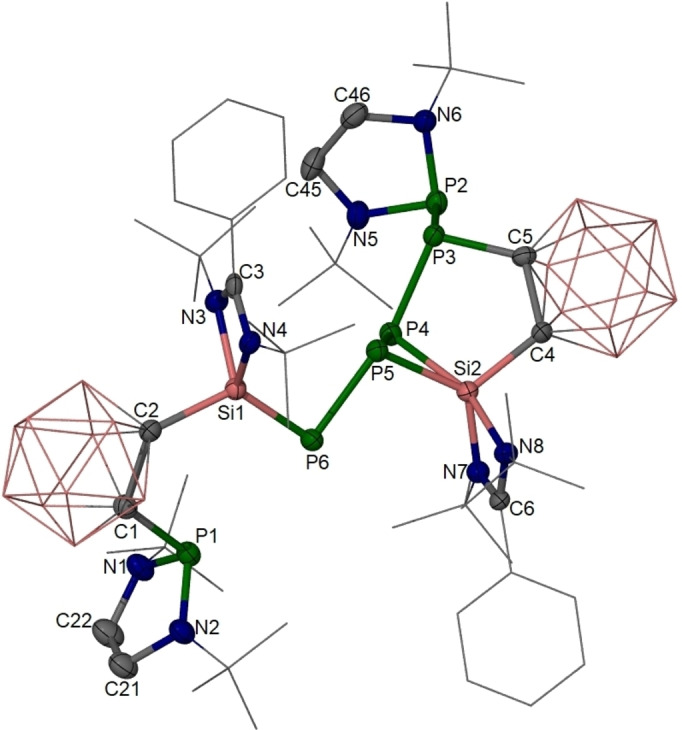
Molecular structure of **2**.^[25]^ Thermal ellipsoids are drawn at 50 % probability level. H atoms and one diethyl ether lattice solvent molecule are omitted for clarity. Selected interatomic distances [Å] and angles [°]: Si1–P6 2.132(1), Si1–C2 1.951(4), Si2–C4 1.935(3), Si2–P4 2.317(1), Si2–P5 2.228(1), P3–C5 1.892(4), P2–P3 2.304(1), P3–P4 2.186(1), P4–P5 2.277(1), P5–P6 2.235(1); C2‐Si1‐P6 121.0(1), Si1‐P6‐P5 91.9(1), N8‐Si2‐P4 161.3(1), N7‐Si2‐C4 114.2(1), N7‐Si2‐P5 127.8(1), C4‐Si2‐P5 117.7(1).

Since one phosphine moiety of the two spend molecules **1** remained unchanged after P_4_ activation, we envisioned that using the bis(silylene) **3** would lead to different products because of the higher reactivity of the silylene moiety. To our surprise, conversion of **3** with P_4_ in the molar ratio of 2 : 1 in THF at ambient temperature afforded the first isolable 1,3‐diphospha‐2,4‐disilabutadiene (Si=P−Si=P)‐containing product **4** in 67 % yields. Akin to the formation of **A** (Figure 1) and **1⋅P_4_** (Scheme 1), we propose the formation of **3⋅P_4_** (Scheme 2) as initial reaction intermediate, which reacts with a second molar equivalent of **3** to afford **4′** and the final product **4**. Fortunately, equimolar reactions of **3** with P_4_ afforded **3⋅P_4_** as the main product along with **4′** and **4** (Figure S11a in Supporting Information). Compounds **3⋅P_4_** and **4′** could be identified by their diagnostic ^31^P{^1^H}‐ and ^1^H‐NMR spectra. The four P atoms in **3⋅P_4_** exhibits three signals in the ^31^P{^1^H}‐NMR spectrum (*δ*_Si= ***P*** PP2Si_=−261.9 ppm, *dt*; *δ*_P ***P*** P2_=170 ppm, *dm*; *δ*_PP ***P*** 2Si_=−48.8 ppm, *dd*) (Figure S11a). Accordingly, the ^1^H‐NMR spectrum of **3⋅P_4_** in THF‐*d*_8_ shows two singlets at *δ*=1.38 and 1.44 ppm for two sets of *t*Bu protons (Figure S12). On the other hand, compound **4′** exhibits a singlet at *δ*=−1.28 ppm for the *t*Bu protons in the ^1^H‐NMR spectrum and one singlet at very high field, *δ*=−407.5 ppm, in the ^31^P NMR spectrum with ^29^Si‐satellites (^1^ *J*_SiP_=84 Hz), which agrees well with the calculated value (^31^P{^1^H}‐NMR: *δ*=−416.3 ppm; Figure S20). The latter ^31^P resonance is up‐field shifted compared with that observed for 2,2,4,4‐tetramesityl‐1,3‐diphospha‐2,4‐disilabicyclo[1.1.0]butane *δ*=−324 ppm; ^1^ *J*_SiP_=77 Hz).^[16]^ Our efforts to isolate **3⋅P_4_** and **4′** failed since even at low temperature they isomerize to **4** along with the formation of small amounts of unidentified species. In line with that, addition of one molar equivalent of **3** to a freshly prepared reaction mixture containing mainly **3⋅P_4_** affords solely **4′** which in turn rearranges to **4** as the final product (Scheme 2). The isomerization of **4′** to **4** is probably due to steric congestion and ring strain in **4′**.

**Scheme 2:**
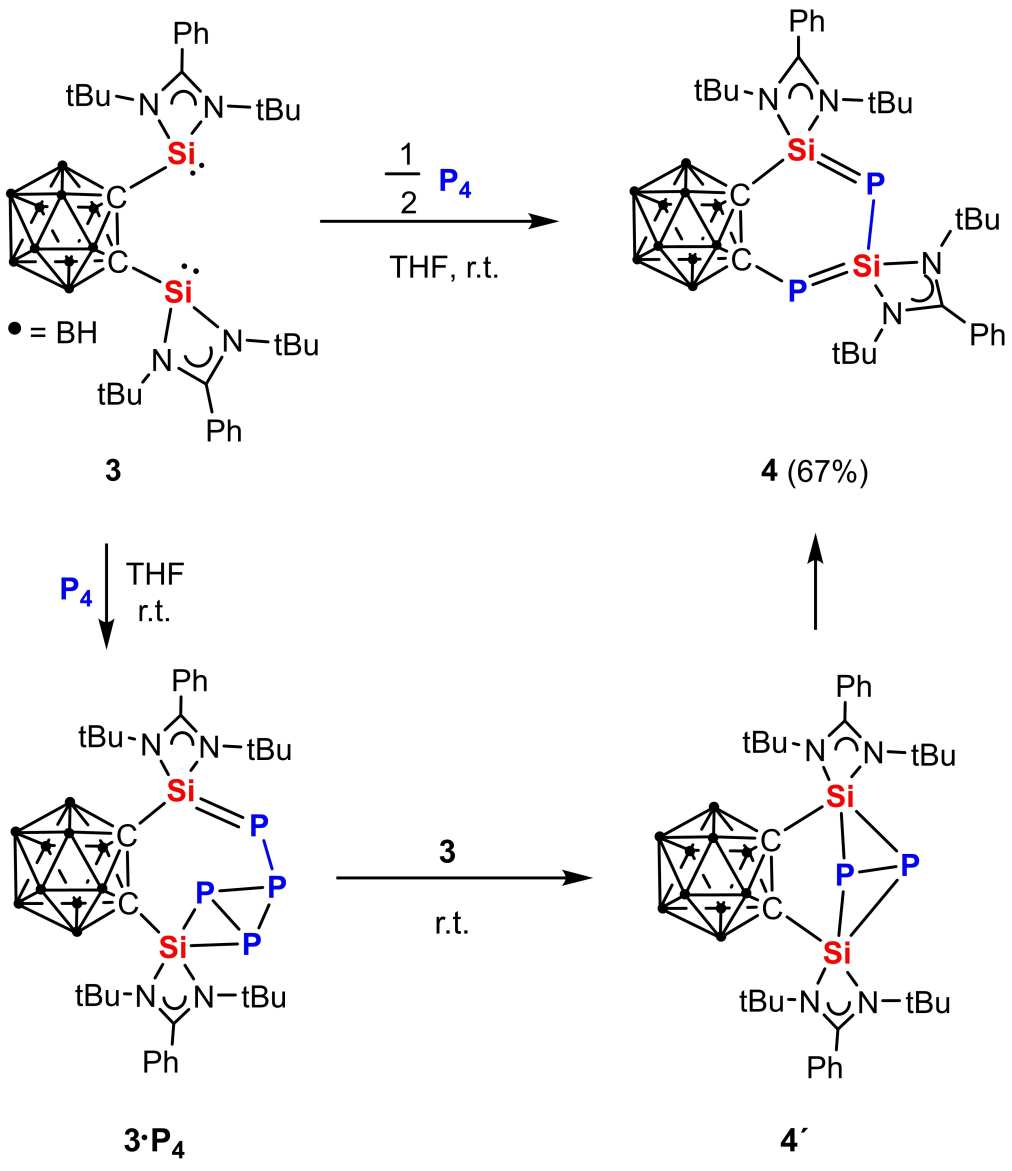
Activation of P_4_ to furnish **4** via **3⋅P_4_** and **4′**.

Compound **4** was characterized NMR‐spectroscopically and by single‐crystal X‐ray diffraction analysis. The ^31^P{^1^H}‐NMR spectrum of **4** exhibits two different P atoms giving rise to two doublets at *δ*=−26.8 ppm (P2, Figure 4) and −269.8 ppm (P1) with ^2^ *J*_PP_=28 Hz. The latter value is close to that in **E** (−282.4 ppm)^[19]^ due to the pronounced zwitterionic Si^+^−P^−^−Si^+^−P^−^ character of the Si=P−Si=P moiety. The low‐field ^31^P resonance at *δ*=−26.8 ppm in **4** for the C‐*P2* atom is most likely due to the strong electron withdrawing character of the *o*‐dicarborane cage. Notably, the ^29^Si{^1^H}‐NMR spectrum of **4** in *d*_8_‐THF exhibits two sets of doublets of doublets at much lower field at *δ*=50.2 (Si1) (Figure 4) and 33.9 ppm (Si2) than that observed in **E** (*δ*=3.7 ppm).^[19]^ Again, this is presumably due to the zwitterionic Si^+^−P^−^−Si^+^−P^−^ character of the Si=P−Si=P moiety.

**Figure 4:**
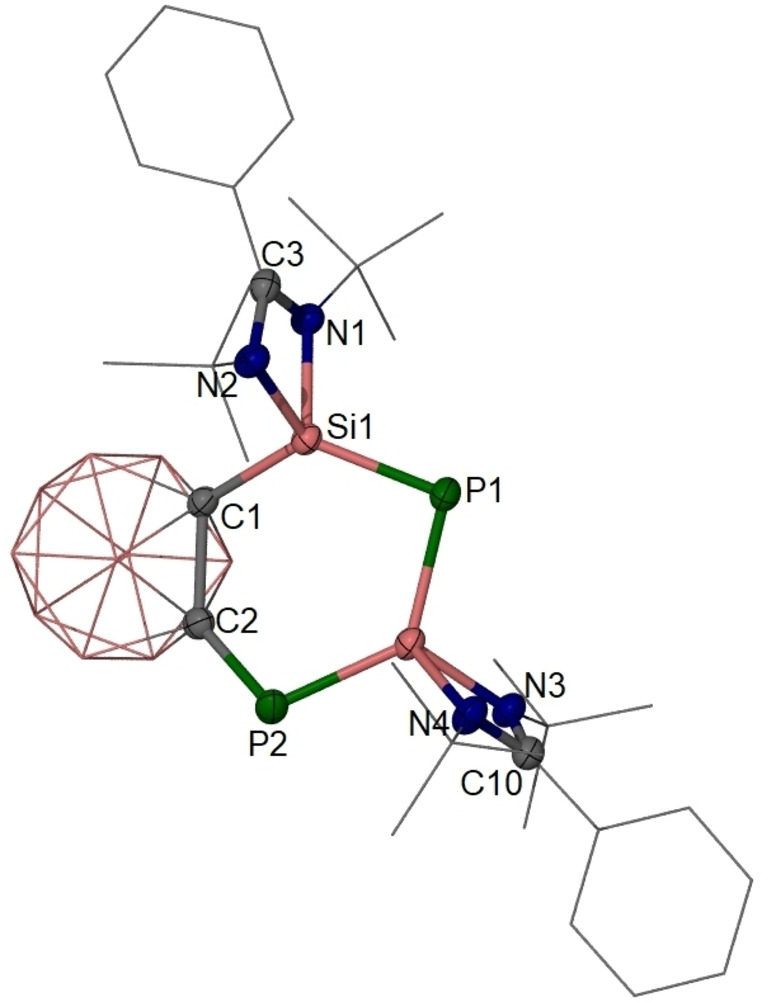
Molecular structure of **4**.^[25]^ Thermal ellipsoids are drawn at 50 % probability level. H atoms are omitted for clarity. Selected interatomic distances [Å] and angles [°]: Si1–C1 1.920(1), Si1–P1 2.118(1), Si2–P1 2.165(1), Si2–P2 2.143(1), P2–C2 1.859(2), C1–C2 1.712(2); C1‐Si1‐P1 125.8(1), Si1‐P1‐Si2 98.2(1), P1‐Si2‐P2 128.0(1), Si2‐P2‐C2 106.1(1), P2‐C2‐C1 126.8(1), C2‐C1‐Si1 120.0(1).

Red single crystals of **4** in the monoclinic space group *I*2_a_ were obtained in THF solution at ambient temperature. Their X‐ray diffraction analysis revealed that one LSi moiety was removed from the *o*‐dicaborandiyl backbone and replaced by a P atom to form a slightly puckered six‐membered C_2_P_2_Si_2_ ring (Figure 4). While both P atoms are two‐coordinate, the two four‐coordinate Si atoms adopt a distorted tetrahedral coordination geometry. The Si1=P1, Si2=P2 and Si2=P1 distance of 2.118(1), 2.143(1) and 2.165(1) Å, respectively, indicate a delocalized zwitterionic Si=P−Si=P structure, which is similar to the Si−P distances in **C** (2.160(1) Å),^[14]^ **D** (2.174(1) Å),^[17]^ **E** (2.130(1) Å),^[19]^ and **2** (2.132(1) Å).

DFT calculations confirmed the presence of a conjugated Si=P−Si=P structure in **4** (Figure 5; Figure S23). The two dominant contributing PIO (Principal Interacting Orbital) pairs[^22^, ^23^] with PBIs (Principal Bond Index) of 0.86 and 0.35, respectively, (Figure 5a) indicate Si1–P1 double bond character, which is in line with the WBI^[24]^ (1.387; Figure S25b). Specifically, the first PIO pair suggests a *σ*‐bonding interaction between the Si1 and the P1 atoms whereas the second PIO pair represents a Si1‐P1 *π* bonding. The analysis of the Si2−P2 bonding interactions gave a similar result (Figure S23c). Notably, the PIO analysis of the P1−Si2 bond (Figure 5b) also revealed two sets of interactions, but the π bond is with PBI of 0.20 weaker than those of the other two Si−P π bonds (0.35 for Si1−P1 and 0.34 for Si2−P2, respectively), in accordance with π‐conjugation between the Si1−P1 and Si2−P2 subunits. The Si^+^−P^−^ bonds in these compounds exhibit a typical polarization character which is supported by the natural population analysis (NPA, Figure S26).

**Figure 5:**
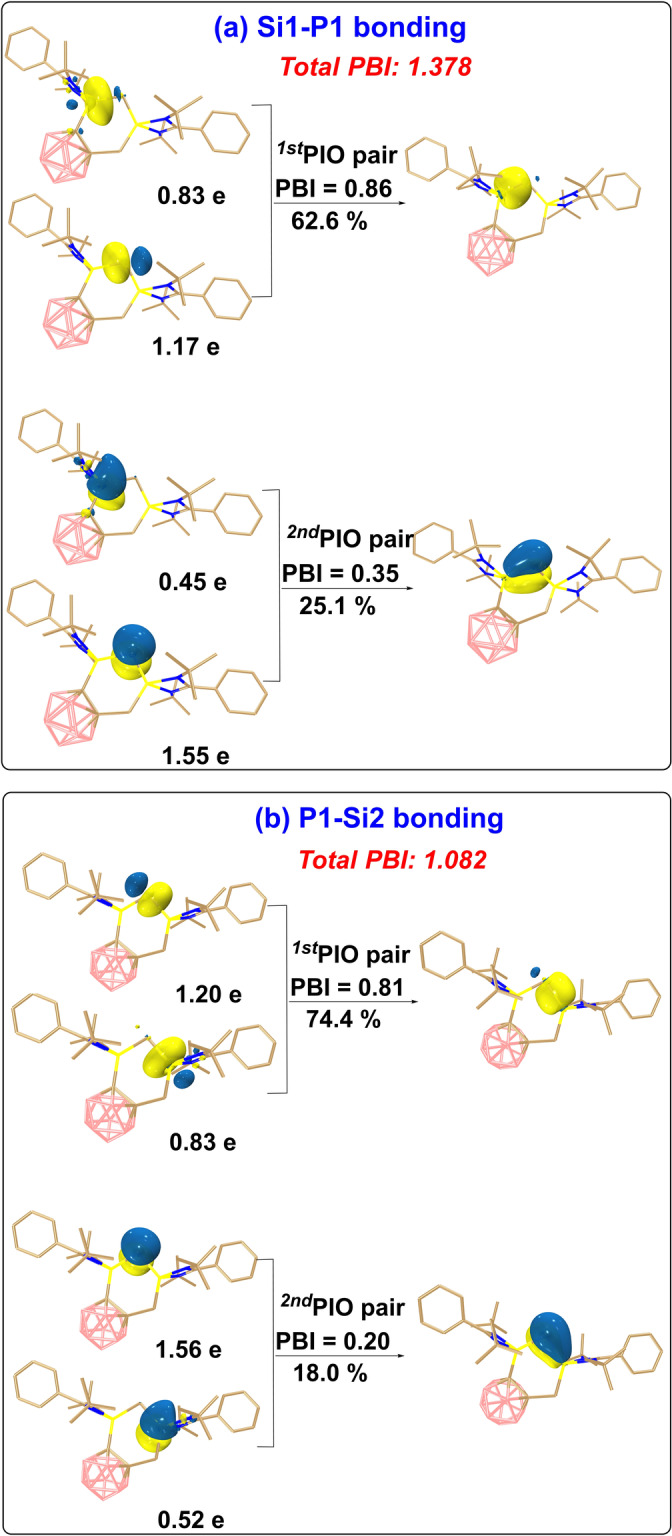
PIO (Principal Interacting Orbital) analysis[^22^, ^23^] on the bonding modes a) between Si1 and P1 atoms and b) between P1 and Si2 atoms in compound **4**. Hydrogen atoms in 3D structures are omitted for clarity. The PIO analysis is performed by cutting the Si−P bonds. The isosurface 0.050 au is plotted.

In summary, new types of metal‐free white phosphorus (P_4_) activation were observed. The phosphine‐silylene‐substituted *o*‐dicarborane **1** is capable to activate white phosphorus in a 2 : 1 molar ratio to form the P_5_ chain‐containing species **2**. In marked contrast, starting from the corresponding bis(silylene) analogue **3**, complete degradation of P_4_ is achieved leading to the isolable Si=P−Si=P compound **4**. For the latter case, two intermediates with P_4_ and P_2_ subunits were observed by multinuclear NMR spectroscopy.

## Conflict of interest

The authors declare no conflict of interest.

## Supporting information

As a service to our authors and readers, this journal provides supporting information supplied by the authors. Such materials are peer reviewed and may be re‐organized for online delivery, but are not copy‐edited or typeset. Technical support issues arising from supporting information (other than missing files) should be addressed to the authors.

Supporting Information

## Data Availability

The data that support the findings of this study are available in the Supporting Information of this article.
