# Supplementary material for: Unexpected White Phosphorus (P4) Activation Modes with Silylene‐Substituted o‐Carboranes and Access to an Isolable 1,3‐Diphospha‐2,4‐disilabutadiene
Source: Angew Chem Int Ed Engl. 2022 May 19;61(28):e202205358. doi: 10.1002/anie.202205358 (PMC9401593; doi:10.1002/anie.202205358)
Supplement: Supplementary file 1 — Supporting Information [file ANIE-61-0-s001.pdf]

Angewandte Chemie
Eine Zeitschrift der Gesellschaft Deutscher Chemiker

Supporting Information

Unexpected White Phosphorus (P_4) Activation Modes with Silylene-Substituted *o*-Carboranes and Access to an Isolable 1,3-Diphospha-2,4-disilabutadiene

*Y. Xiong, S. Dong, S. Yao, J. Zhu, M. Driess**

Table of Content

A. Experimental Section……………………………………….………………....………..…..3

A1 General Considerations……………………………………….……….……….…..………… 3

A2 Single-Crystal X-ray Structure Determination………………………...…..….……………..... 3

A3 Synthesis and Characterization…………………………………..……………..……….. ….3

A4 Details of the Single Crystal X-ray Diffraction Analyses…………………….……..…….….16

B Computational Section…………………………..……..…………………….….…..….……...24

C References…...…………………………………………….……….….….……....……...38

A. Experimental Section

A1. General Considerations

All experiments and manipulations were carried out under dry nitrogen using standard Schlenk techniques or in an MBraun inert atmosphere dry box containing an atmosphere of purified N_2. Solvents were deoxygenated and dried by standard methods, saturated with purified N_2 and freshly distilled prior to use. The precursor compounds Silylene-Phosphine **1** [1] and Bis(silylene) **3** [2] were prepared according to our previous work. The ^{1}H, ^{13}C, ^{31}P, ^{11}B, ^{29}Si-NMR spectra were recorded on Brucker ARX200, AV400, AV500 spectrometers referenced to residual solvent signals as internal standards (^{1}H and $^{13}C\{^{1}H\}$) or with an external reference ($SiMe_4$ for ^{29}Si). Abbreviations: *s* = singlet; *d* = doublet; *t* = triplet; *sept* = septet; *m* = multiplet; *br* = broad. IR spectra were measured with a Nicolet iS5 FT-IR Spectrometer from the company of Thermo Scientific. Elemental analyses were performed on a FlashEA 1112 CHNS Analyzer. Melting points were measured on a Stuart SMP30 melting point apparatus.

A2. Single-Crystal X-ray Structure Determinations

Crystals were each mounted on a glass capillary in per-fluorinated oil and measured in a cold N_2 flow. The data of **2** and **4** were collected on an Oxford Diffraction Supernova, Single source at offset, Atlas at 150 K (Cu- Kα-radiation, λ = 1.5418 Å). The structures were solved by direct method and refined on F2 with the SHELX-97 [3] software packages. The positions of the H atoms were calculated and considered isotropically according to a riding model. CCDC Deposition Numbers 2165356 (**2**) and 2165357 (**4**), contain the Supplementary Crystallographic Data for this paper.

A3. Synthesis and Characterization

Compound $(LSiP)_2P_4$ (2): 50 mL Et_2O was added to a mixture of **1** (0.54 g, 0.89 mmol) and P_4 (0.054 g, 0.44 mmol) at room temperature. The reaction mixture was stirred for 16 h. Yellow precipitate were formed which was isolated by filtration. The collected yellow

precipitate of **2** amounted to 0.50 g (0.75 mmol, 84%). The qualified crystals for x-ray diffraction analysis were obtained from the filtrate at room temperature. M.p. 213 °C (decomp.); **^{1}H NMR** (200.13 MHz, THF-d_8, 298 K): δ (ppm) = 1.34 (*s*, 18 H; P=Si-NC(C*H*$_3$)$_3$), 1.42 (*s*, 9 H; NC(C*H*$_3$)$_3$), 1.45 (*s*, 9 H; NC(C*H*$_3$)$_3$), 1.46 (*s*, 9 H; NC(C*H*$_3$)$_3$), 1.48 (*s*, 9 H; NC(C*H*$_3$)$_3$), 1.54 (*s*, 18 H; P-NC(C*H*$_3$)$_3$), 1.90 – 3.20 (*br*., 20 H; B*H*), 7.50 – 7.99 (*m*, 10 H; *Ph*); **^{11}B{^{1}H} NMR** (64.21 MHz, THF-d_8, 298 K): *δ* (ppm) = -2.50 ppm (*br*.); **^{13}C{^{1}H} NMR** (100.61 MHz, THF-d_8, 298 K): *δ* (ppm) = 29.3 (*d*, $^{3}J_{PC}$ = 10 Hz, PNC(*C*H$_3$)$_3$), 29.4 (*d*, $^{3}J_{PC}$ = 10 Hz, PNC(*C*H$_3$)$_3$), 29.5 (d, $^{3}J_{PC}$ = 10 Hz, PNC(*C*H$_3$)$_3$), 30.5, 30.6, 30.9, 31.3 (*s*, SiNC(*C*H$_3$)$_3$), 33.9 (*d*, $^{3}J_{PC}$ = 10 Hz, PNC(*C*H$_3$)$_3$), 46.2 (*d*, $^{2}J_{PC}$ = 6 Hz, PN*C*H$_2$), 46.8 (*d*, $^{2}J_{PC}$ = 6 Hz, PN*C*H$_2$), 47.1 (*d*, $^{2}J_{PC}$ = 8 Hz, PN*C*H$_2$), 47.8 (*d*, $^{2}J_{PC}$ = 8 Hz, PN*C*H$_2$), 53.7 (*d*, $^{2}J_{PC}$ = 13 Hz, PN*C*Me$_3$), 54.4 (*d*, $^{2}J_{PC}$ = 21 Hz, PN*C*Me$_3$), 54.7 (*d*, $^{2}J_{PC}$ = 26 Hz, PN*C*Me$_3$), 55.2 (*d*, $^{2}J_{PC}$ = 26 Hz, PN*C*Me$_3$), 55.3, 55.6, 56.0, 56.2 (*s*, SiN*C*Me$_3$), 79.5 (*br.* cage-*C*), 81.0 (*m*, cage-*C*), 84.2 (*m*, cage-*C*), 99.3 (*d*, $^{1}J_{PC}$ = 138 Hz, cage-*C*), 126.8, 127.7, 127.8, 128.0, 128.7, 130.0 (*Ph*), 130.2 (quaternary *Ph*), 130.3, 131.0 (*Ph*), 133.0 (quaternary *Ph*), 177.6, 178.8 ppm (N*C*N); **^{31}P{^{1}H} NMR** (81.01 MHz, THF-d_8, 298 K): *δ* (ppm) = 135.5 (*ddd*, $^{1}J_{P2P3}$ = 291 Hz, J_{P2P6} = 16Hz, $^{2}J_{P2P4}$ = 10Hz, *P2*), 106.8 (*ddd*, J_{P1P5} = 420 Hz, J_{P1P6} = 16Hz, J_{P1P3} = 10Hz, *P1*), 40.0 (*ddddd*, $^{1}J_{P3P4}$ = 369Hz, $^{1}J_{P3P2}$ = 291 Hz, J_{P3P6} = 131Hz, J_{P3P1} = 10Hz, J_{P3P5} = 5Hz, *P3*), -145.8 (*dddd*, $^{1}J_{P4P3}$ = 369Hz, $^{1}J_{P4P5}$ = 72 Hz, $^{2}J_{P4P2}$ = 10Hz, $^{2}J_{P4P6}$ = 10Hz, *P4*), -191.8 (*dddd*, J_{P5P1} = 420Hz, $^{1}J_{P5P6}$ = 189Hz, $^{1}J_{P5P4}$ = 72Hz, $^{2}J_{P5P3}$ = 5Hz, *P5*), -225.5 (*ddddd*, $^{1}J_{P6P5}$ = 189Hz, J_{P6P3} = 131Hz, J_{P6P1} = $^{2}J_{P6P2}$ = 16Hz, $^{2}J_{P6P4}$ = 10Hz, *P6*); **^{29}Si{^{1}H} NMR** (79.49 MHz, THF-d_8, 298 K): *δ* (ppm) = 39.2 (*ddd,* $^{1}J_{Si1P6}$ = 256 Hz, $^{2}J_{Si1P5}$ = 32 Hz, $^{3}J_{Si1P1}$ = 9 Hz, P=*Si*), -68.0 ppm (*td*, $^{1}J_{Si2P4}$ = $^{1}J_{Si2P5}$ = 72Hz, $^{2}J_{Si2P}$ = 25Hz, *Si2*); Elemental analysis calcd (%) for $C_{54}H_{110}B_{20}N_8P_6Si_2 \cdot Et_2O$ (1403.85 g/mol) C 49.62, H 8.62, N 7.98, found: C 49.28, H 8.70, N 7.85. **IR** (cm^{-1}): 2967(*m*), 2555(*m*), 1526(*w*), 1475(*m*), 1396 (*s*), 1364(*s*), 1245(*m*), 1195(*vs*), 1093(*w*), 1066(*s*), 1023(*w*), 1000(*w*), 964(*m*), 930(*w*), 875(*w*), 814(*w*), 788(*m*), 761(*s*), 732(*s*), 707(*s*), 669(*w*), 652(*s*), 623(*s*).

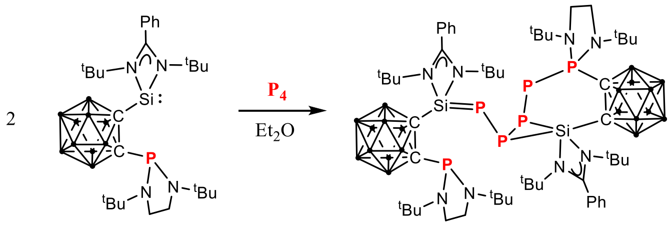

Scheme S1. Synthesis of **2.**

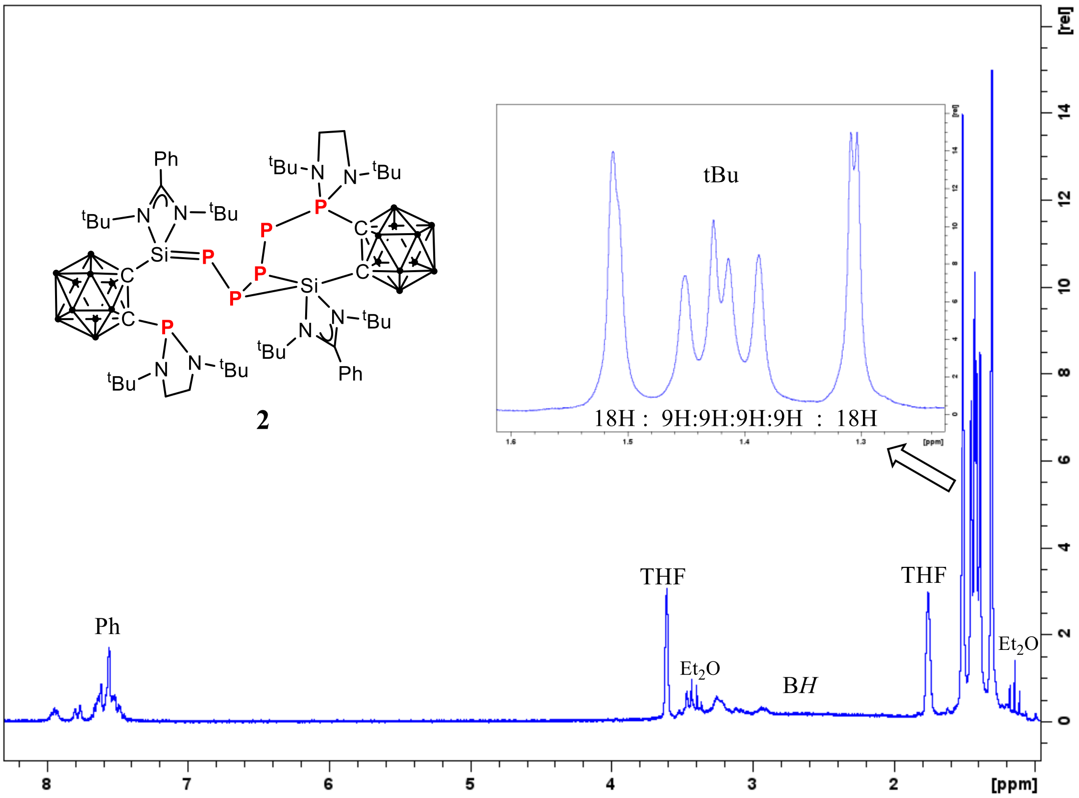

Figure S1. ^{1}H NMR spectrum of **2** (200.13 MHz, THF-d_8, 298 K).

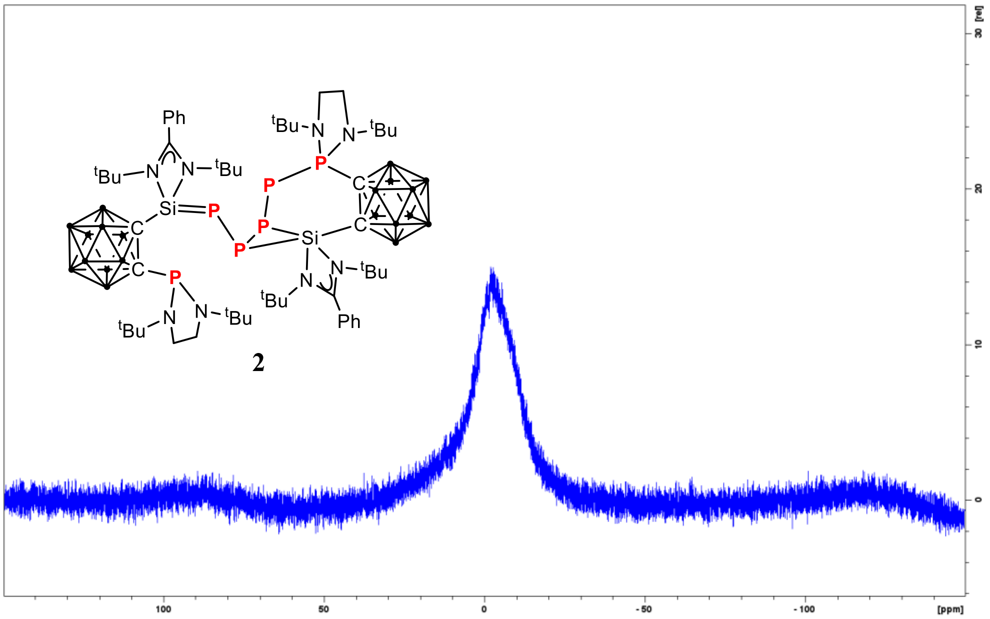

Figure S2. ^{11}B NMR spectrum of **2** (64.21 MHz, THF-d_8, 298 K).

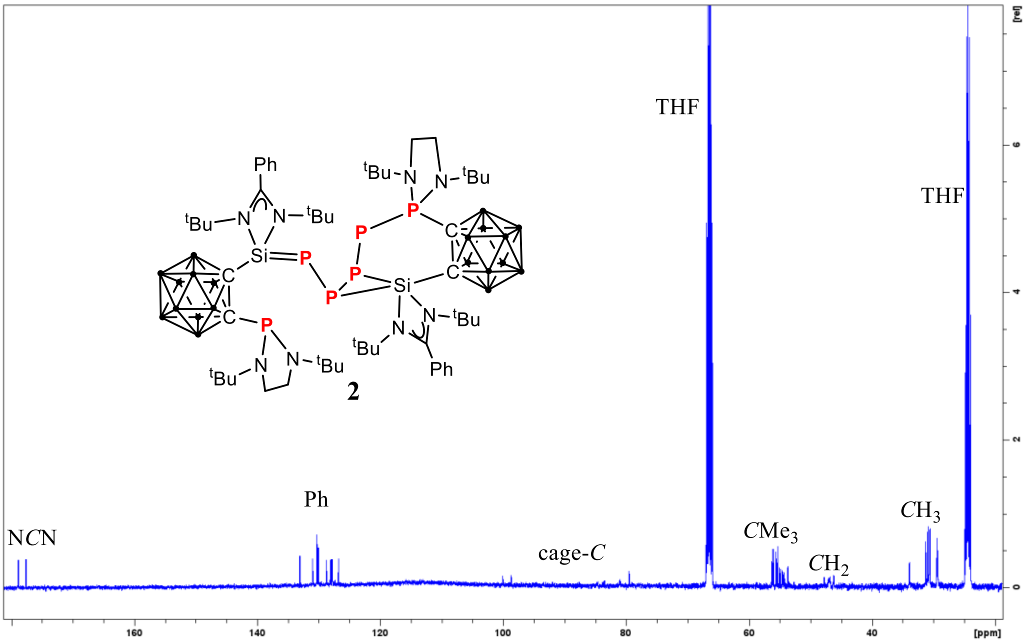

Figure S3. ^{13}C{^{1}H} NMR spectrum of **2** (100.61 MHz, THF-d_8, 298 K).

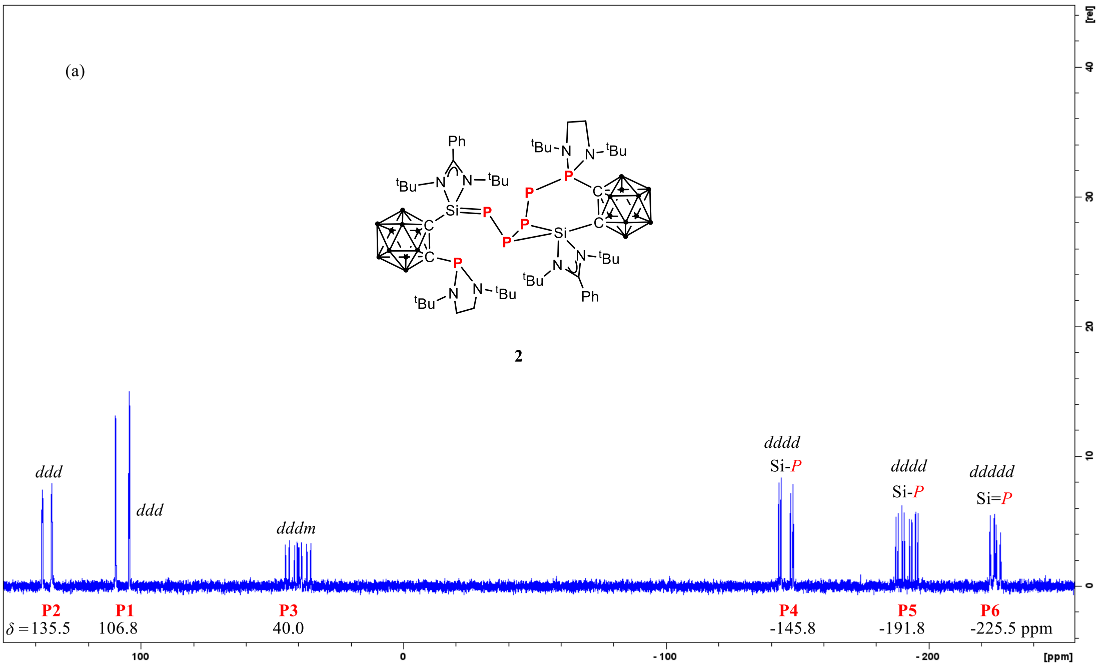

Figure S4 (**a**) $^{31}P\{^1H\}$ NMR spectrum of **2** (81.01 MHz, THF-d_8, 298 K).

(b)

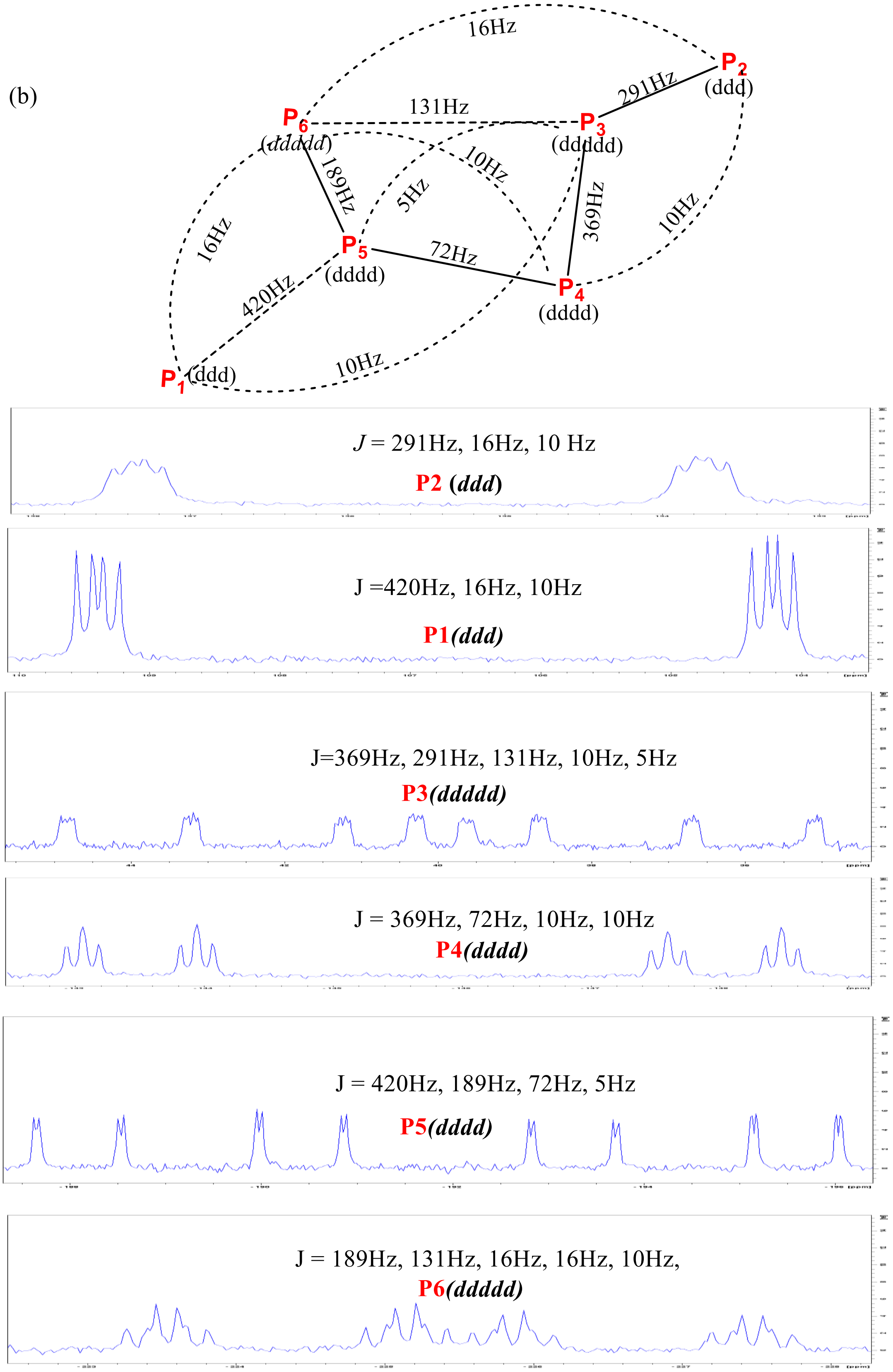

Figure S4 (**b**) Enlarged NMR signals for each P atom in **2**.

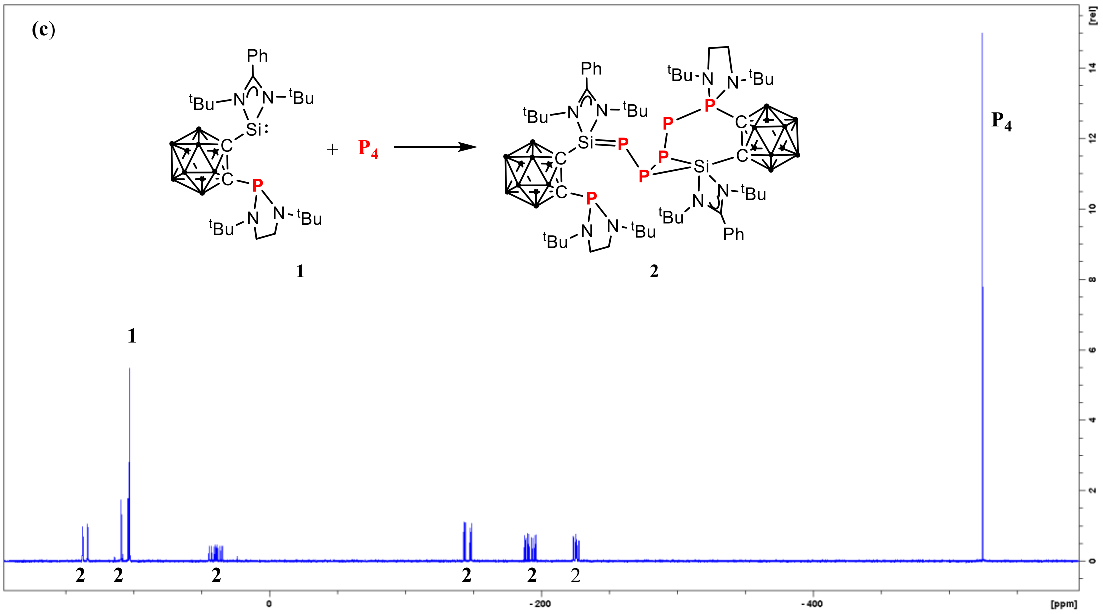

Figure S4 (c) $^{31}P\{^1H\}$ NMR spectrum of the reaction mixture of **1**, P_4, and **2**. No signals for any intermediate could be observed.

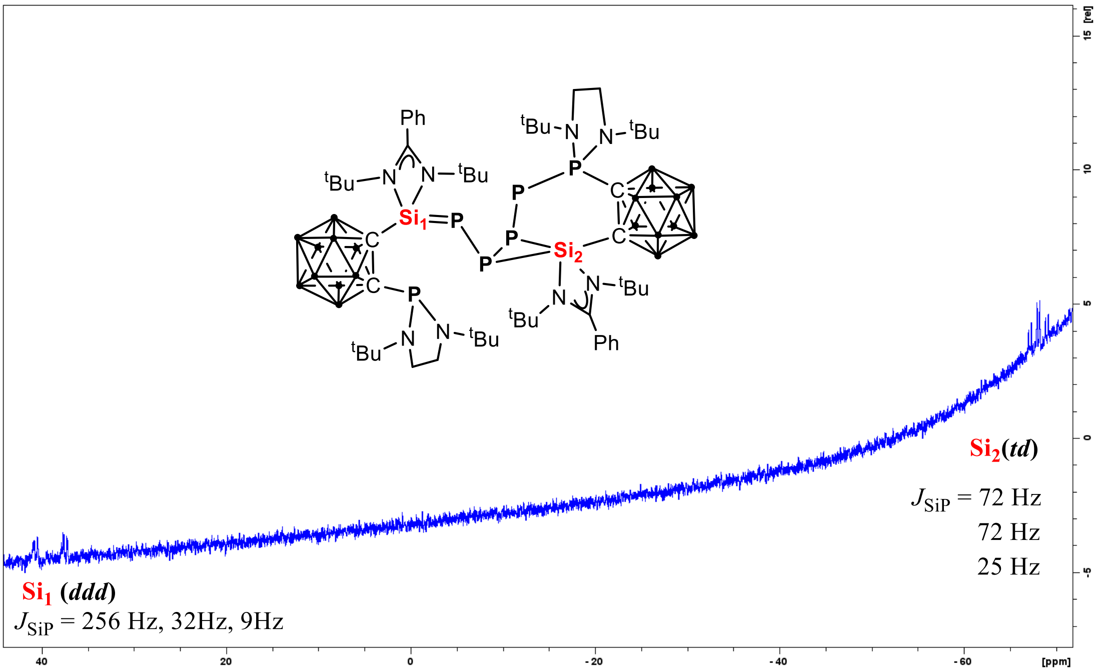

Figure S5. $^{29}Si\{^1H\}$ NMR spectrum of **2** (79.49 MHz, THF-d_8, 298 K).

Compound $CBSi_2P_2$ (4): 25 mL THF was added to a mixture of the bis(silylene) **3** (0.24 g, 0.36 mmol) and P_4 (0.022 g, 0.18 mmol) at room temperature. The reaction mixture was stirred overnight. The solvent was concentrated to 10 mL. After two weeks red crystals of **4** at room temperature were formed which are qualified for X-ray diffraction analysis. The collected crystals amounted to 0.17 g (0.24 mmol, 67%). M.p. 130 °C (decomp.); **^{1}H NMR** (200.13 MHz, THF-d_8, 298 K): δ(ppm) = 1.25 (*s*, 18 H, C*H*$_3$), 1.41 (*s*, 18 H, C*H*$_3$), 7.48 – 7.78 (*m*, 10 H, *Ph*); **^{11}B{^{1}H} NMR** (64.21 MHz, THF-d_8, 298 K): δ (ppm) = -8.1 ppm; **^{13}C{^{1}H} NMR** (50.32 MHz, THF-d_8, 298 K): δ(ppm) = 30.91, 30.94 (C(CH_3)$_3$), 54.8, 56.4(*C*Me_3), 127.8, 127.9, 128.0, 128.2, 128.4, 128.5, 128.7, 129.0 (*Ph*), 130.1 (quaternary *Ph*), 130.2, 131.2 (*Ph*), 131.9 (quaternary *Ph*), 172.8, 180.0 (N*C*N); **^{31}P{^{1}H} NMR** (81.01 MHz, THF-d_8, 298 K): δ (ppm) = -26.8 (*d*, $^{2}J_{PP}$ = 28 Hz, Si*P*C), -269.8 (*d*, $^{1}J_{PP}$ = 28 Hz, *SiP*Si); **^{29}Si{^{1}H} NMR** (79.49 MHz, THF-d_8, 298 K): δ (ppm) = 50.2 (*dd*, $^{1}J_{SiP}$ = 93.6Hz, 150Hz, P*Si*P), 33.9 ppm (*dd*, $^{1}J_{SiP}$ = 146Hz, $^{3}J_{SiP}$ = 3.9Hz, C*Si*P); Elemental analysis calcd (%) for $C_{32}H_{56}B_{10}N_4Si_2P_2$: (723.04 g/mol) C 53.16, H 7.81, N 7.75, found: C 52.97, H 7.71, N 7.79. **IR** (cm^{-1}): 2971(*m*), 2556(*m*), 1517(*w*), 1474(*m*), 1444(*m*), 1392(*vs*), 1364 (*s*), 1266(*m*), 1193(*s*), 1069(*s*), 1021(*m*), 924(*w*), 877(*w*), 792(*m*), 764(*s*), 730(*s*), 706(*vs*), 679(*w*), 631(*s*), 615(*m*), 607(*s*).

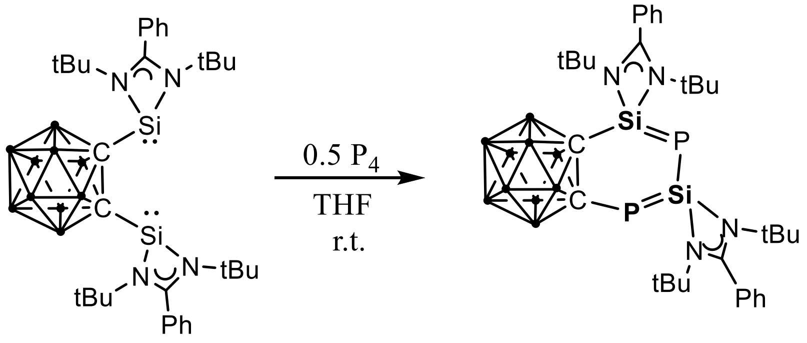

3 **4** (67%)

Scheme S2. Synthesis of **4.**

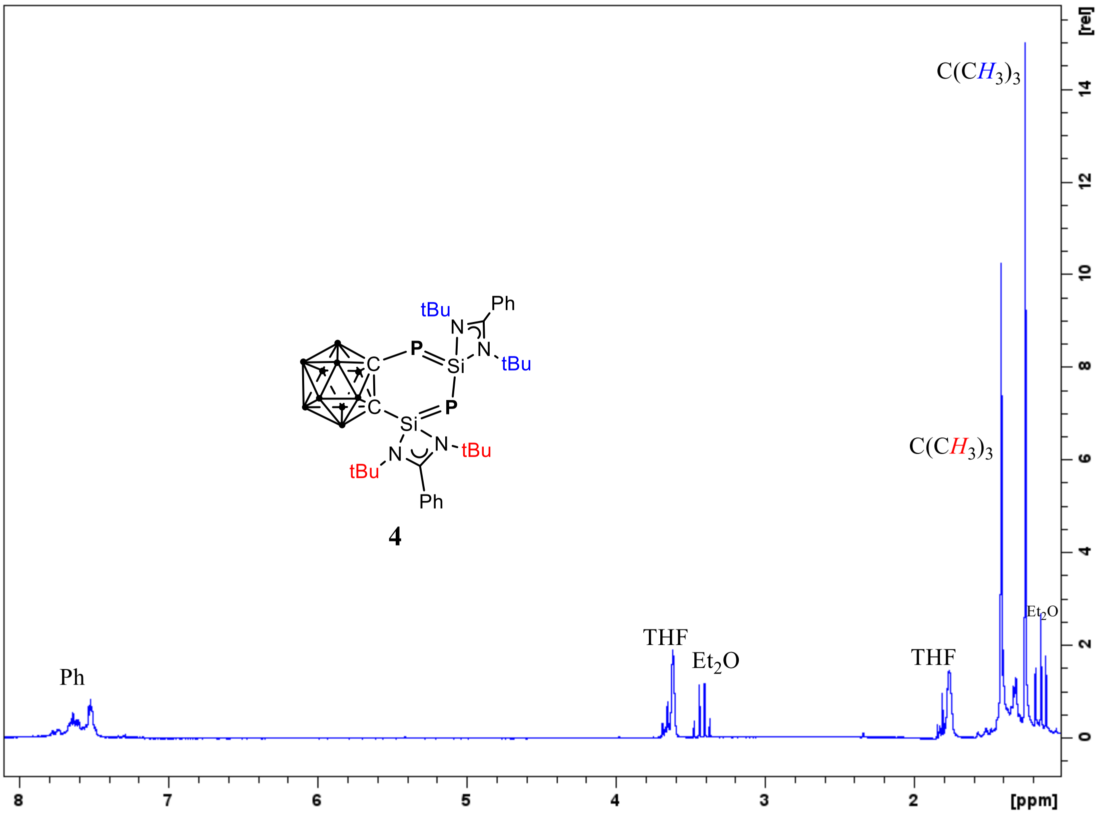

Figure S6. ^{1}H NMR spectrum of **4** (200.13 MHz, THF-d_8, 298 K).

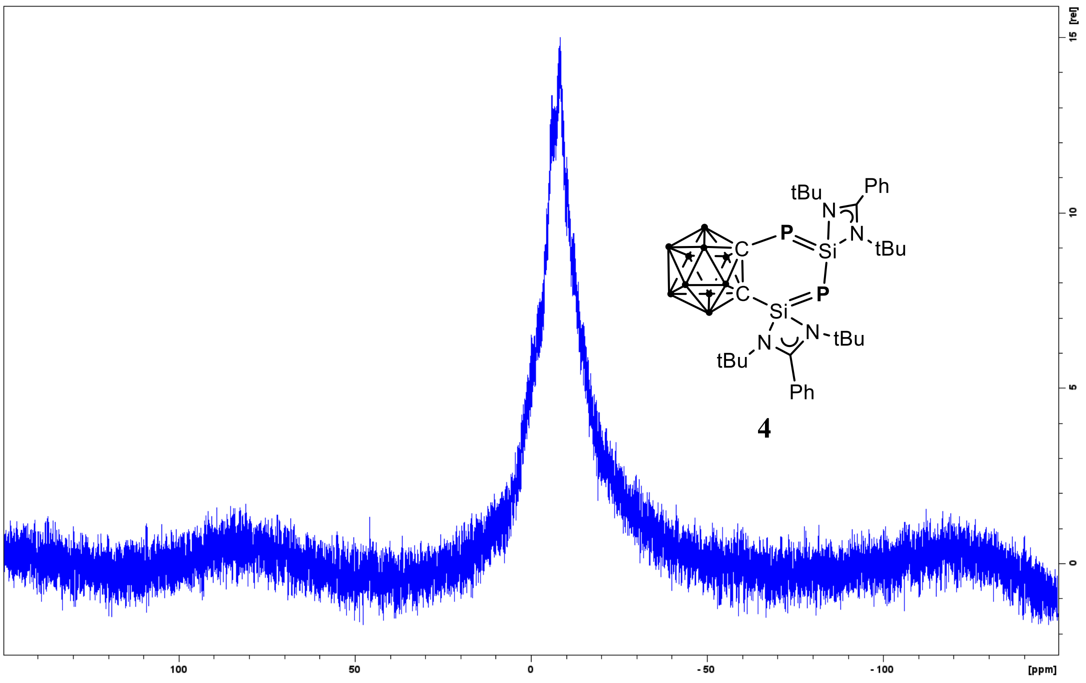

Figure S7. $^{11}B\{^{1}H\}$ NMR spectrum of **4** (64.21 MHz, THF-d_8, 298 K).

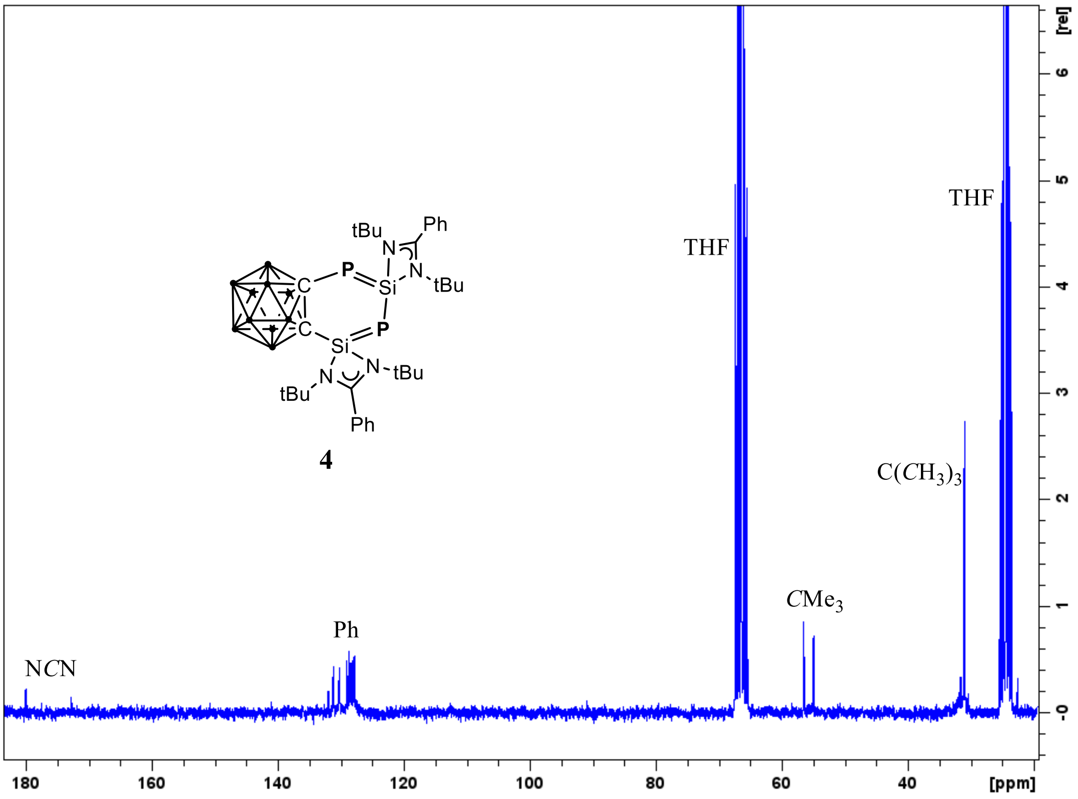

Figure S8. ^{13}C{^{1}H} NMR spectrum of **4** (50.32 MHz, THF-*d*8, 298 K).

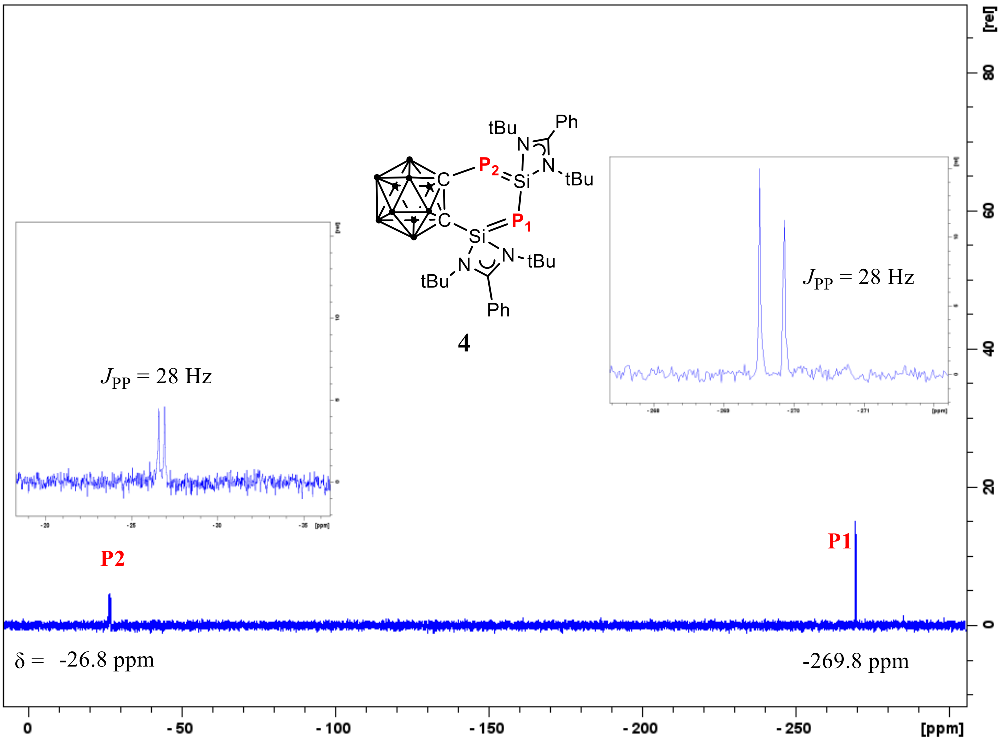

Figure S9. ^{31}P{^{1}H} NMR spectrum of **4** (81.01 MHz, THF-*d*8, 298 K).

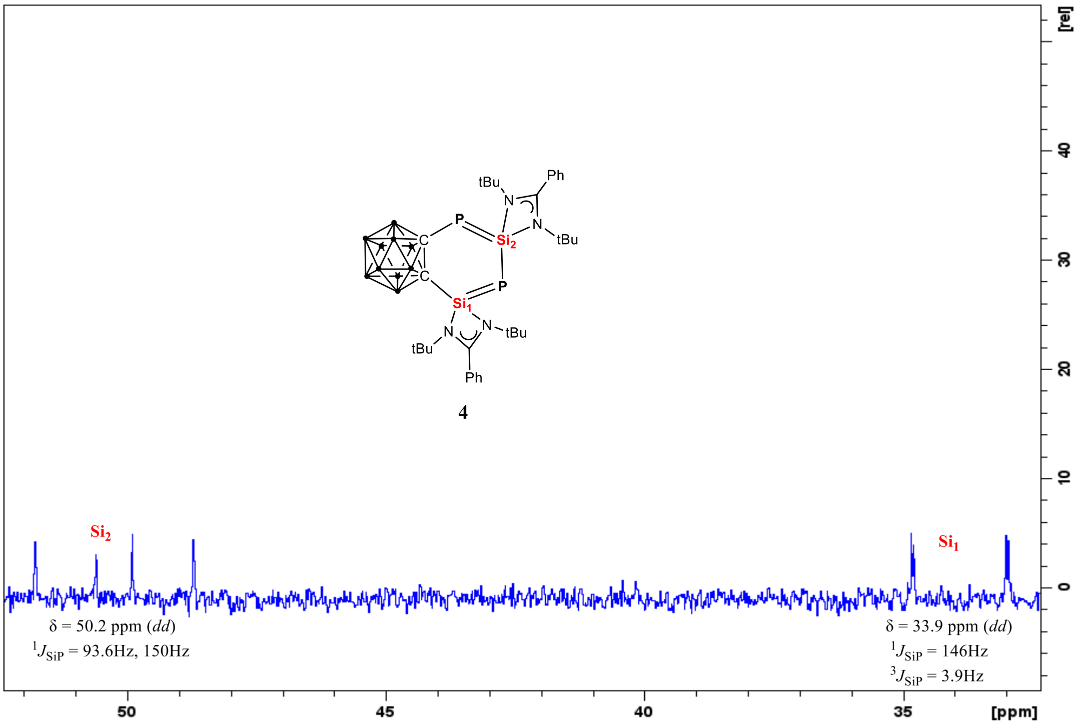

Figure S10. ^{29}Si{^{1}H} NMR spectrum of **4** (79.49 MHz, THF-d_8, 298 K).

Compounds 3·P$_4$ and 4´: A solution of **3** (0.46 g, 0.69 mmol) in THF (15 mL) was added dropwise to a solution of white phosphorus (0.085 g, 0.69 mmol) in THF (30 mL) at -20 °C under stirring. After stirring for 5 h the reaction mixture was concentrated to about 10 mL and stored at -20 °C. After several days no crystals of **3·P$_4$**, **4´** or **4** could be obtained. By further concentration of the solution or alternation of the solvent the isolation of any compound failed.

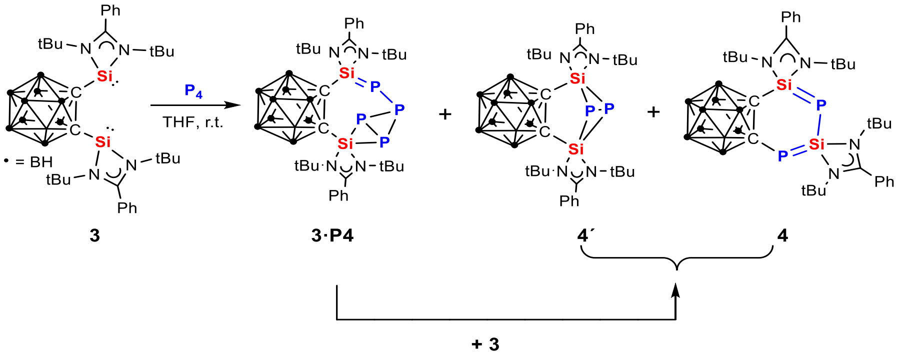

Scheme S3. Formation of **3·P$_4$**, **4´**, and **4**

Conversion of 3·P_4 to 4´and 4 in NMR scale reaction: A solution of P_4 (0.0021 g, 0.013 mmol) in 0.30 ml *d*$_8$-THF in a nmr tube and a solution of bis(silylene) **3** (0.011 g, 0.017 mmol) in 0.20 mL THF-*d*$_8$ in an another nmr tube were prepared. The latter was added dropwise to the P_4 solution in THF-*d*$_8$ at room temperature. The ^{1}H and ^{31}P{^{1}H} NMR measurement showed that a mixture of **3·P_4, 4´** and **4** was obtained (Fig. S11(a)).

Conversion of 3·P_4 to 4´and 4 in the presence of 3: When another equivalent of **3** (0.011g, 0.017 mmol) was added to the above reaction solution in the nmr tube, the signals of **3·P_4** disappeared immediately, and the amounts of **4´** and **4** increased drastically (Fig. S11(b)).

In the absence of 3: The isomerization of **3·P_4** gave **4´** and **4** slowly. Meanwhile **4´** converted also to **4** (^{31}P{^{1}H} NMR in Fig. S11(c), ^{1}H NMR in Fig. S12).

For **3·P_4**: **^{1}H NMR** (200.13 MHz, THF-*d*$_8$, 298 K): δ (ppm) = 1.38 (*s*, tBu), 1.44 (*s*, tBu); **^{31}P{^{1}H} NMR** (81.01 MHz, THF-*d*$_8$, 298 K): δ (ppm) = 170 (*dm*, $^1J_{PP}$ = 105 Hz, P*P*P2), -48.8 (*dd*, $^1J_{PP}$ = 128 Hz, $^2J_{PP}$ = 20 Hz, 2*P,* PP*P2*), -261.9 (*td*, $^1J_{PP}$ =128 Hz, $^1J_{PP}$ = 105Hz, *P*=Si). For **4´**: **^{1}H NMR** (200.13 MHz, THF-*d*$_8$, 298 K): δ(ppm) = 1.28 (*s*) (tBu); **^{31}P{^{1}H} NMR** (81.01 MHz, THF-*d*$_8$, 298 K): δ (ppm) = -407.5 (*s*, $^1J_{SiP}$ = 84 Hz). For **4**: **^{1}H NMR** (200.13 MHz, THF-*d*$_8$, 298 K): δ(ppm) = 1.25 (s, tBu), 1.41(s, tBu); **^{31}P{^{1}H} NMR** (81.01 MHz, THF-*d*$_8$, 298 K): δ (ppm) = -26.8 (d, ^2J =28 Hz), -269.8 ppm (d, ^2J = 28 Hz).

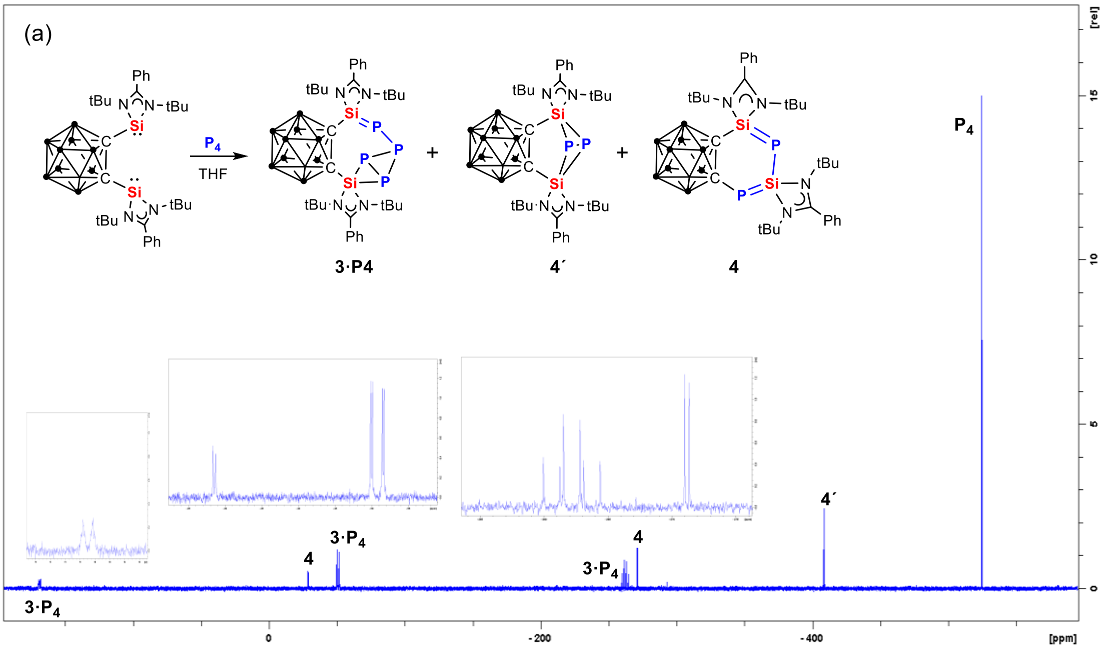

Figure S11(a) $^{31}P\{^{1}H\}$ NMR spectrum of the1:1 molar ratio reaction mixture of **3·P$_4$**, **4´**, **4,** and remained P_4 in d_8-THF.

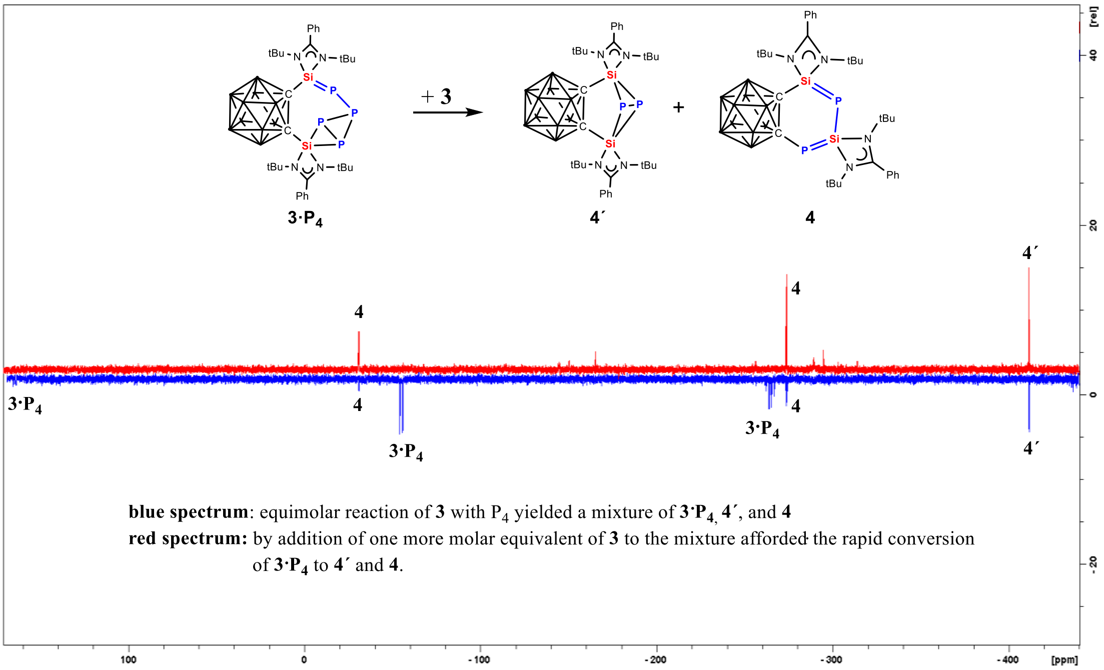

Figure S11(b) $^{31}P\{^{1}H\}$ NMR Spectra in d_8-THF. Conversion of **3·P$_4$** to **4´** and **4**.

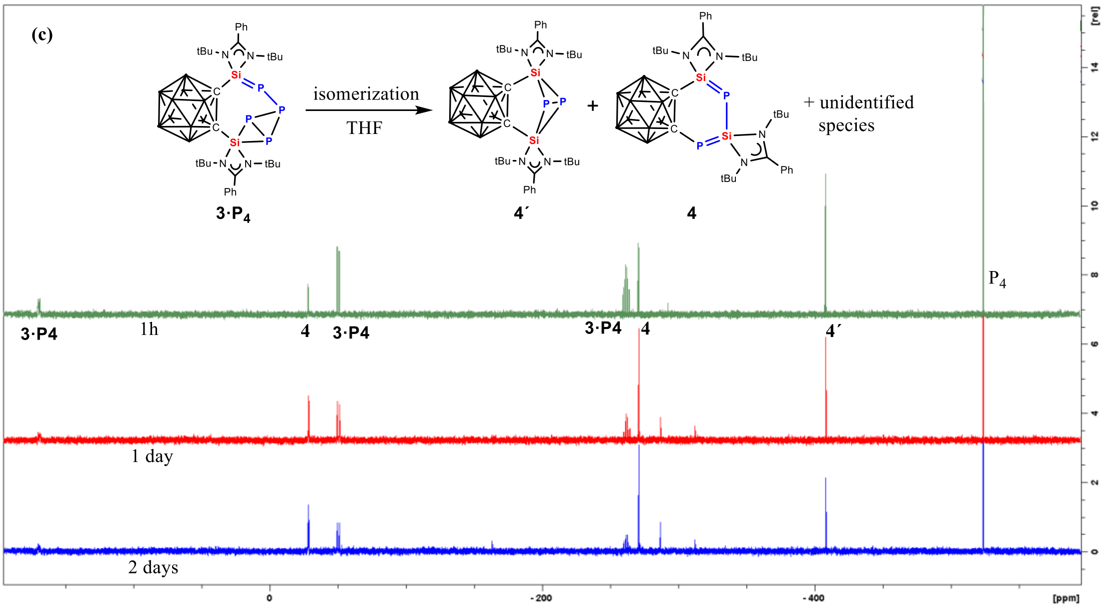

Figure S11(c) $^{31}P\{^1H\}$ NMR spectra. Isomerization of **3·P$_4$** to give **4´, 4** and small amount of unidentified species.

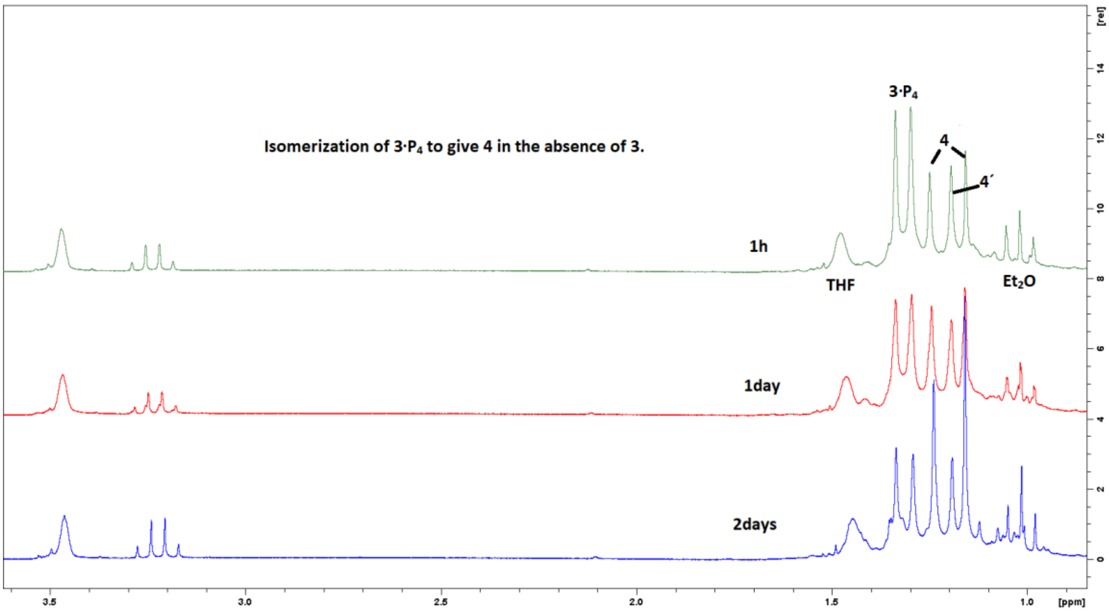

Figure S12. ^1H NMR spectrum in d_8-THF. Isomerization of **3·P$_4$** to give **4´**, **4** and small amount of unidentified species.

A4. Details of the Single Crystal X-ray Diffraction Analyses

Table S1. Crystal data and structure refinement for CB(**SiP**)$_2$**P**$_4$ (**2**).

Empirical formula	C58 H120 B20 N8 O P6 Si2

Formula weight	1403.81	
Temperature	150(2) K	
Wavelength	1.54184 Å	
Crystal system	Triclinic	
Space group	P-1	
Unit cell dimensions	a = 13.7027(6) Å	α= 72.719(4)°.
	b = 16.5891(8) Å	β= 85.815(4)°.
	c = 19.2581(9) Å	γ = 76.777(4)°.
Volume	4069.2(3) $Å^3$	
Z	2	
Density (calculated)	1.146 Mg/m^3	
Absorption coefficient	1.820 mm^{-1}	
F(000)	1500	
Crystal size	0.230 x 0.160 x 0.120 mm^3	
Theta range for data collection	2.403 to 67.500°.	
Index ranges	-16<=h<=16, -19<=k<=19, -23<=l<=22	
Reflections collected	29082	
Independent reflections	14659 [R(int) = 0.0788]	
Completeness to theta = 67.500°	100.0 %	
Absorption correction	Semi-empirical from equivalents	
Max. and min. transmission	1.00000 and 0.83605	
Refinement method	Full-matrix least-squares on F^2	
Data / restraints / parameters	14659 / 0 / 882	
Goodness-of-fit on F^2	1.023	
Final R indices [I>2sigma(I)]	R1 = 0.0558, wR2 = 0.1095	
R indices (all data)	R1 = 0.1133, wR2 = 0.1415	
Extinction coefficient	n/a	
Largest diff. peak and hole	0.498 and -0.478 e.$Å^{-3}$	

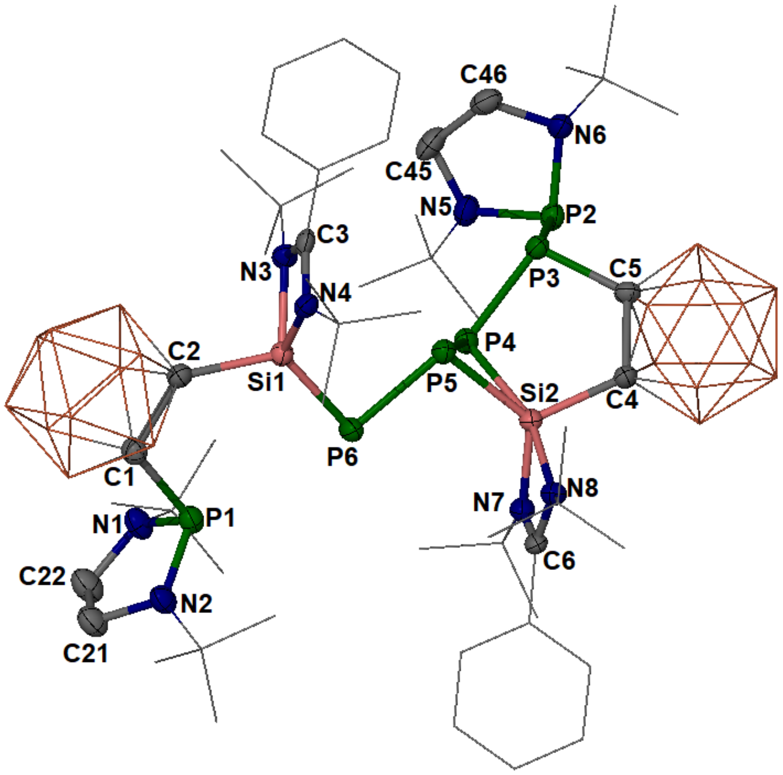

Figure S13. Molecular structure of CB(**SiP**)$_2$**P**$_4$ (**2**). Thermal ellipsoids are drawn at 50% probability level. Hydrogen atoms and one Et_2O molecule are omitted for clarity.

Table S2. Crystal data and structure refinement for CB(**SiP**)$_2$**P**$_4$ (**2**).

P5-Si2	2.228(1)
P5-P6	2.235(1)
P5-P4	2.277(1)
P3-C5	1.892(4)
P3-P4	2.186(1)
P3-P2	2.304(1)
Si1-N4	1.836(3)
Si1-N3	1.840(3)
Si1-C2	1.951(4)
Si1-P6	2.132(1)
P4-Si2	2.317(1)

Si2-N7	1.821(3)
Si2-C4	1.935(3)
Si2-N8	1.945(3)
P2-N5	1.690(3)
P2-N6	1.718(3)
P1-N2	1.671(3)
P1-N1	1.699(3)
P1-C1	1.953(4)
N8-C6	1.310(4)
N7-C6	1.359(4)
N3-C3	1.338(4)
N4-C3	1.338(4)
N6-C46	1.484(5)
N1-C22	1.488(4)
C5-C4	1.688(4)
N5-C45	1.462(5)
C46-C45	1.533(5)
C21-C22	1.508(5)

Si2-P5-P6	100.7(1)
Si2-P5-P4	61.9(1)
P6-P5-P4	100.9()
C5-P3-P4	101.1(1)
C5-P3-P2	99.8(1)
P4-P3-P2	101.2(1)
N4-Si1-N3	71.7(1)
N4-Si1-C2	102.2(1)
N3-Si1-C2	105.3(1)
N4-Si1-P6	123.3(1)
N3-Si1-P6	122.3(1)
C2-Si1-P6	121.0(1)
Si1-P6-P5	91.9(1)
P3-P4-P5	96.0(1)
P3-P4-Si2	98.3(1)
P5-P4-Si2	58.0(1)
N7-Si2-C4	114.2(1)
N7-Si2-N8	69.7(1)
C4-Si2-N8	99.6(1)

N7-Si2-P5	127.8(1)
C4-Si2-P5	117.7(1)
N8-Si2-P5	106.4(1)
N7-Si2-P4	107.2(1)
C4-Si2-P4	98.4(1)
N8-Si2-P4	161.3(1)
P5-Si2-P4	60.1(1)
N7-Si2-C6	35.6(1)
C4-Si2-C6	112.8(1)
N8-Si2-C6	34.2(1)
P5-Si2-C6	120.5(1)
P4-Si2-C6	138.6(1)
N5-P2-N6	94.7(1)
N5-P2-P3	103.2(1)
N6-P2-P3	93.2(1)
N2-P1-N1	93.9(1)
N2-P1-C1	102.6(1)
N1-P1-C1	99.4(2)
C6-N8-Si2	89.2(2)
C3-N3-Si1	90.5(2)
C3-N4-Si1	90.6(2)
C21-N2-P1	112.6(2)
N4-C3-N3	107.1(3)
N4-C3-Si1	53.5(2)
N3-C3-Si1	53.7(2)
C22-N1-P1	111.4(2)
C1-C2-Si1	125.0(2)
C2-C1-P1	117.1(2)
C4-C5-P3	117.2(2)
C5-C4-Si2	116.1(2)
N8-C6-N7	107.6(3)
N8-C6-Si2	56.6(2)
N7-C6-Si2	51.3(2)
N2-C21-C22	106.3(3)
N1-C22-C21	109.3(3)

Symmetry transformations used to generate equivalent atoms:

Table S3. Bond lengths [Å] and angles [°] for CB**Si**$_2$P$_2$ (**4**).

Empirical formula	C32 H56 B10 N4 P2 Si2	
Formula weight	723.02	
Temperature	150(2) K	
Wavelength	1.54184 Å	
Crystal system	Monoclinic	
Space group	I2/a	
Unit cell dimensions	a = 25.7508(3) Å	α= 90°.
	b = 13.2404(2) Å	β= 99.8730(10)°.
	c = 26.2696(4) Å	γ = 90°.
Volume	8824.0(2) Å^3	
Z	8	
Density (calculated)	1.088 Mg/m^3	
Absorption coefficient	1.607 mm^{-1}	
F(000)	3072	
Crystal size	0.490 x 0.260 x 0.200 mm^3	
Theta range for data collection	3.415 to 67.496°.	
Index ranges	-30<=h<=28, -15<=k<=14, -30<=l<=31	
Reflections collected	16967	
Independent reflections	7946 [R(int) = 0.0215]	
Completeness to theta = 67.496°	99.8 %	
Absorption correction	Semi-empirical from equivalents	
Max. and min. transmission	1.00000 and 0.41759	
Refinement method	Full-matrix least-squares on F^2	
Data / restraints / parameters	7946 / 0 / 463	
Goodness-of-fit on F^2	1.080	
Final R indices [I>2sigma(I)]	R1 = 0.0381, wR2 = 0.1016	
R indices (all data)	R1 = 0.0432, wR2 = 0.1050	
Extinction coefficient	n/a	
Largest diff. peak and hole	0.456 and -0.362 e.Å^{-3}	

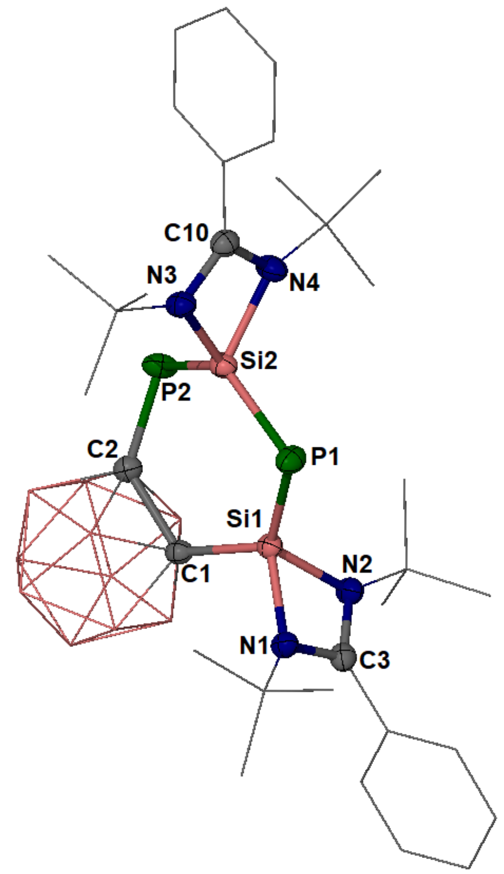

Figure S14. Molecular structure of for CB**Si**$_2$P$_2$ (**4**). Thermal ellipsoids are drawn at 50% probability level. Hydrogen atoms are omitted for clarity.

Table S4. Bond lengths [Å] and angles [°] for CB**Si$_2$P$_2$** (**4**).

Si1-N1	1.818(1)
Si1-N2	1.837(1)
Si1-C1	1.919(2)
Si1-P1	2.118(1)
P1-Si2	2.165(1)
Si2-N4	1.844(1)
Si2-N3	1.860(1)
Si2-P2	2.143(1)
P2-C2	1.859(2)
N1-C3	1.349(2)
N2-C3	1.334(2)
N3-C10	1.332(2)
N4-C10	1.340(2)

C2-C1	1.712(2)
N1-Si1-N2	72.1(1)
N1-Si1-C1	105.6(1)
N2-Si1-C1	105.5(1)
N1-Si1-P1	112.1(1)
N2-Si1-P1	122.1(1)
C1-Si1-P1	125.8(1)
Si1-P1-Si2	98.2(1)
N4-Si2-N3	70.7(1)
N4-Si2-P2	103.7(1)
N3-Si2-P2	116.3(1)
N4-Si2-P1	115.1(1)
N3-Si2-P1	108.6(1)
P2-Si2-P1	128.0(1)
C2-P2-Si2	106.1(1)
C3-N1-Si1	90.8(1)
C3-N2-Si1	90.4(1)
C10-N3-Si2	91.1(1)
C10-N4-Si2	91.5(1)
C1-C2-P2	126.8(1)
C2-C1-Si1	120.1(1)
N2-C3-N1	106.6(1)

B. Computational Section

Computational details. All the calculations were performed using the Gaussian 16 software package.[4] Geometry optimization of the compounds was conducted at the TPSS-D3BJ[5] density functional theory level, according to the best agreement with the metric data from X-ray structure analyses (Table S1). The Def2-SVP[6] basis set is used to describe C, N, H and B atoms, whereas ma-TZVP[7,8] basis set is used to Si and P atoms. In addition, frequency calculations are carried out at the same level of theory to confirm the stationary points are minima with no imaginary frequencies. Furthermore, the B97-2[9]/Def2-TZVP[10] method is used to calculate the ^{31}P NMR chemical shifts, where the solvent effect (solvent = THF) is taken into account by SMD model. The calculated ^{31}P absolute shielding constants are converted to ^{31}P NMR chemical shifts, with 85% water solution of H_3PO_4 as reference. Here we use $\sigma(H_3PO_4)$ = 328.35 ppm suggested by Jameson *et al.*[11] Viewing of optimized structures and rendering of molecular orbitals were performed using the program *CYLview*[12] and *VMD*[13], respectively. NMR spectra were drawn in the *Multiwfn* program[14]. Density matrices of natural atomic orbitals (NAO) used for the natural population analysis (NPA) were obtained using the *NBO7.0* program[15].

B1. For Compound 2

NMR analysis

According to the DFT calculation, the resonances located at 177.6 ($\sigma_{exp.}$ = 135.5), 137.3 ($\sigma_{exp.}$ = 106.8), 68.7 ($\sigma_{exp.}$ = 40.0), -122.7 ($\sigma_{exp.}$ = -145.8), -177.6 ($\sigma_{exp.}$ = -191.8), and -200.0 ($\sigma_{exp.}$ = -225.5) ppm of **2** are assigned to the P2, P1, P3, P4, P5 and P6 atoms, respectively (Figure S15).

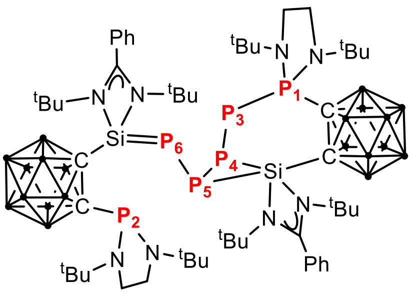

$(SiP)_2P_4$ (2)

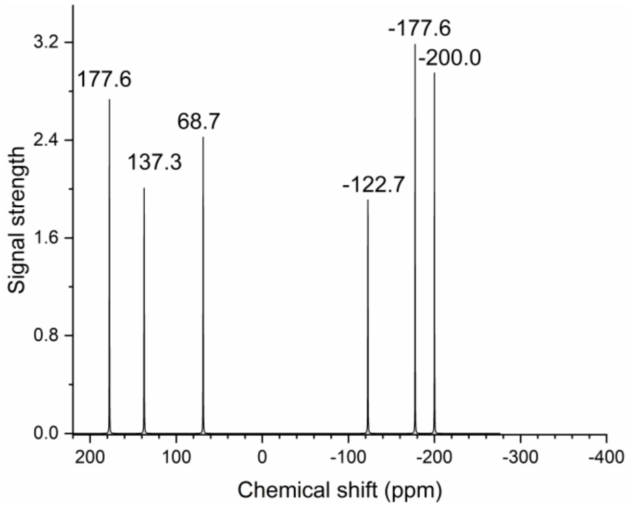

Figure S15. DFT-calculated ^{31}P NMR spectrum of **2**.

The nature of Si-P bonds in compound $(SiP)_2P_4$ (2)

Table S5 Key distances (Å) of experimental and DFT-optimized structures of compound **2**.[a]

Functional	Exp.	TPSS
Si1-P6	2.132	2.128
P6-P5	2.235	2.231
P5-P4	2.277	2.301
P5-Si2	2.228	2.197
P4-Si2	2.317	2.326
P4-P3	2.186	2.187
P3-P2	2.304	2.315
RD(%)[a]	0	0.5

[a] RD = $\frac{\sum_{i=1}^{n}\frac{|BL(DFT) - BL(Exp)|}{BL(Exp)} * 100\%}{n}$, BL means bond length.

The Si1-P6 bond in **2** can be supported by the two dominating contributing PIO pairs with PBIs of 0.83 and 0.39, respectively (Figure 16, b), and the Wiberg bond index of 1.351. The first and second PIO pairs correspond to a σ type donation and a π-backdonation, respectively. In addition, the σ type bond of between Si2 and P5 atoms is also confirmed by both the PIO analysis with total close-to-one PBI value of 0.90 and close-to-one Wiberg bond index of 0.899 (Figure S17). Compared to the Si2-P5 bond, the Si2-P4 σ type bond is slightly weaker, according to a little smaller PBI value of 0.70 (Figure S18) and Wiberg bond index of 0.70.

(a)

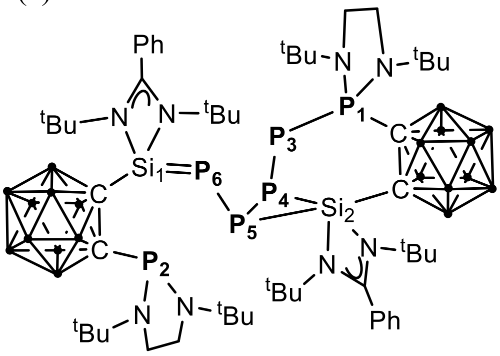

$(SiP)_2P_4$ (2)

(b) Si1-P6

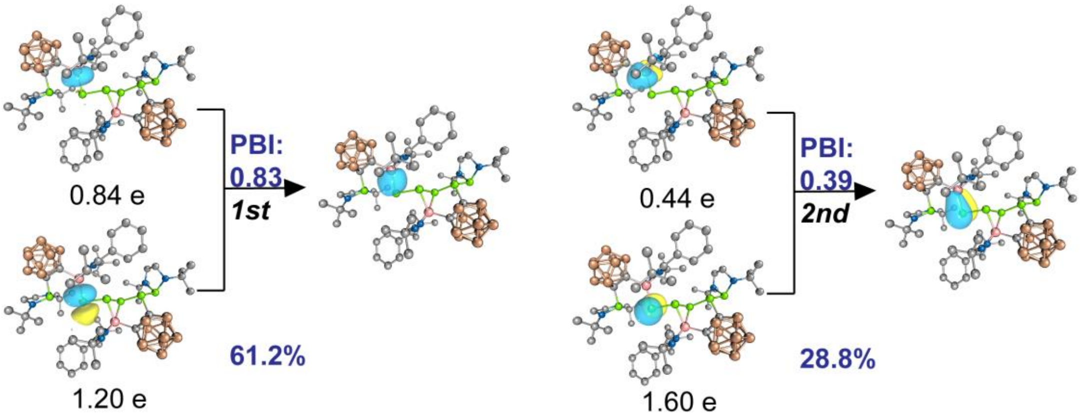

Figure S16. PIO analysis on the bonding modes of Si-P in compound **2**. Hydrogen atoms in 3D structures are omitted for clarity. The PIO analysis is performed to the Si and P atoms. Each PIO pair leads to a bonding PIMO. The PBI quantifies the strength of the interaction. The total PBI value of Si1-P6 bonds is 1.35. The isosurface 0.050 au is plotted.

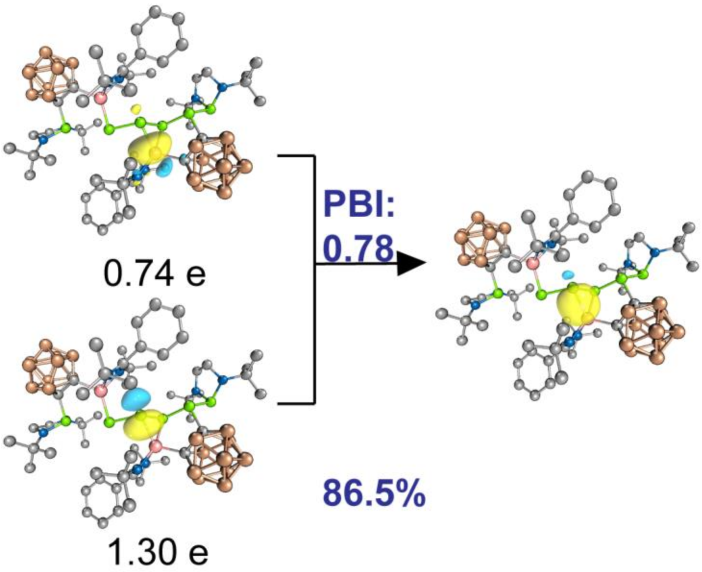

Figure S17. PIO analysis on the bonding modes of Si2-P5 in compound **2**. Hydrogen atoms in 3D structures are omitted for clarity. The PIO analysis is performed to the Si and P atoms. Each PIO pair leads to a bonding PIMO. The PBI quantifies the strength of the interaction. The total PBI value of Si2-P5 bond is 0.90. The isosurface 0.050 au is plotted.

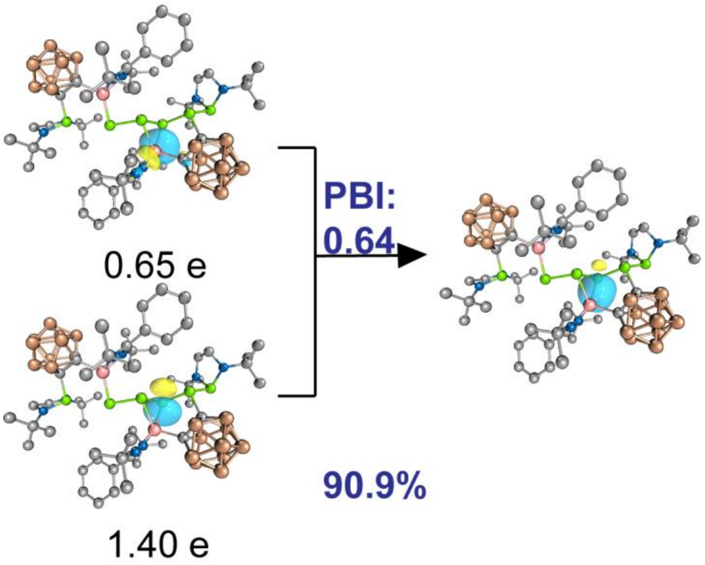

Figure S18. PIO analysis on the bonding modes of Si2-P4 in compound **2**. Hydrogen atoms in 3D structures are omitted for clarity. The PIO analysis is performed to the Si and P atoms. Each PIO pair leads to a bonding PIMO. The PBI quantifies the strength of the interaction. The total PBI value of Si2-P4 bond is 0.70. The isosurface 0.050 au is plotted.

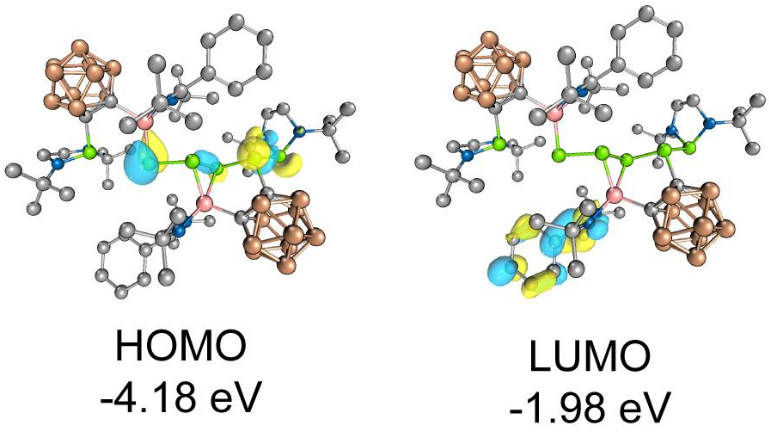

Figure S19. HOMO and LUMO orbitals of the compound **2**. Hydrogen atoms in 3D structures are omitted for clarity.

B2. For Compound 4´

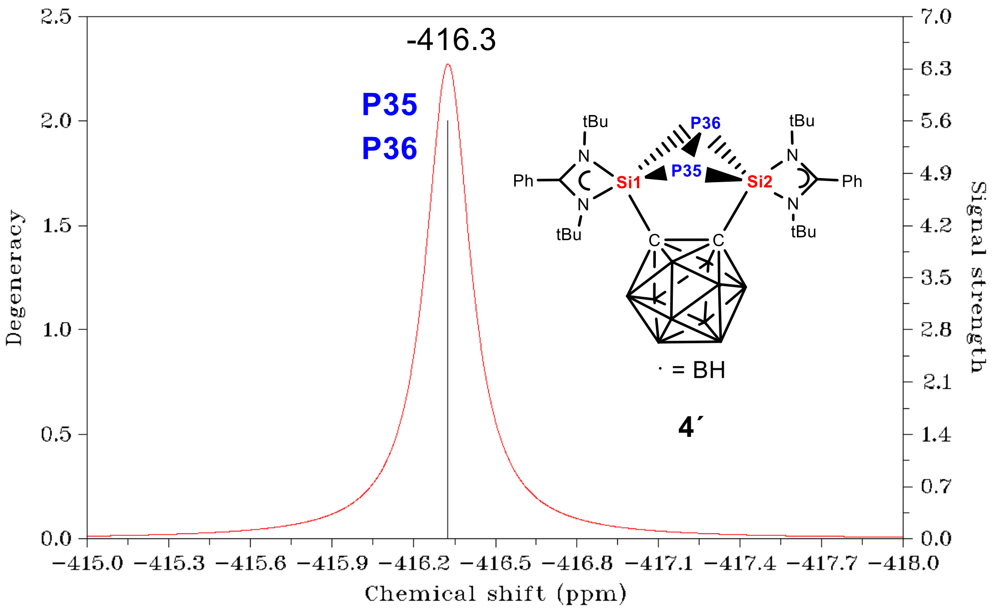

Figure S20. Calculated ^{31}P NMR Spectrum of **4´**.

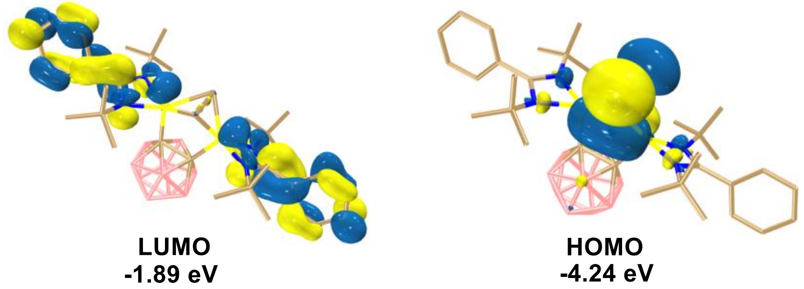

Figure S21. HOMO and LUMO orbitals of the compound **4´**. Hydrogen atoms in 3D structures are omitted for clarity. The isosurface 0.030 au is plotted.

B3. For Compound 4

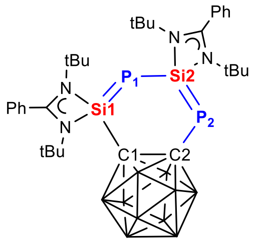

4

Table S6 Key distances (Å) of experimental and DFT-optimized structures of compound **4**.[a]

Functional	Exp.	TPSS
C1-Si1	1.919	1.914
Si1-P1	2.118	2.118
P1-Si2	2.165	2.178
Si2-P2	2.143	2.144
P2-C2	1.859	1.866
C2-C1	1.712	1.705
RD(%)[a]	0	0.55

[a] RD = $\frac{\sum_{i=1}^{n}\frac{|BL(DFT)-BL(Exp)|}{BL(Exp)}*100\%}{n}$, BL means bond length.

NMR analysis. According to the DFT calculation, the peaks located at -13.0 and -265.3 ppm of **4** are assigned to the P2 and P1 atoms, respectively (Figure S22).

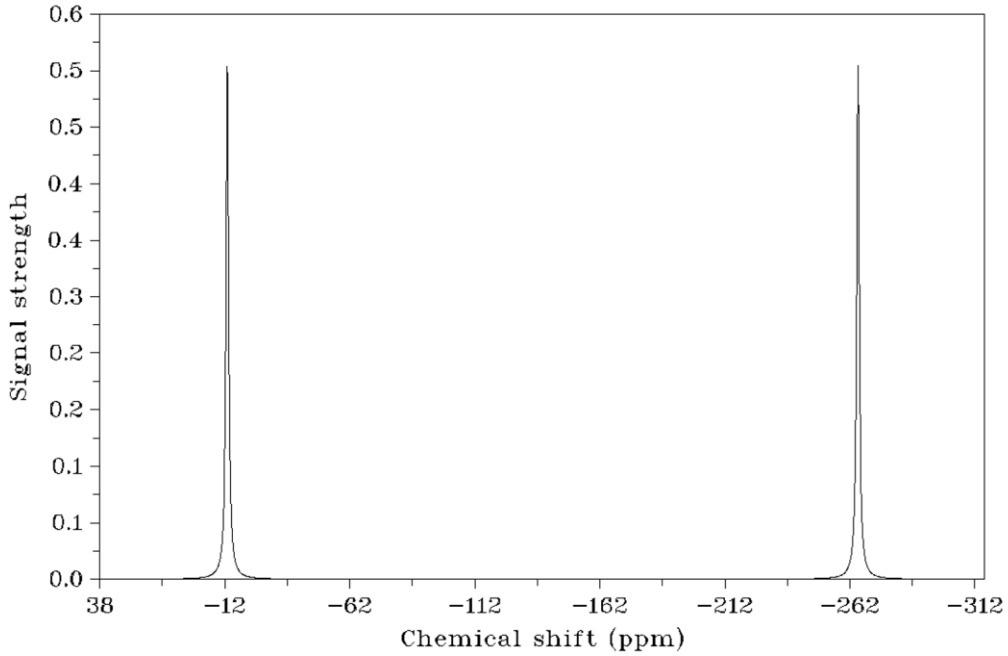

Figure S22. Calculated ^{31}P NMR Spectrum of **4**.

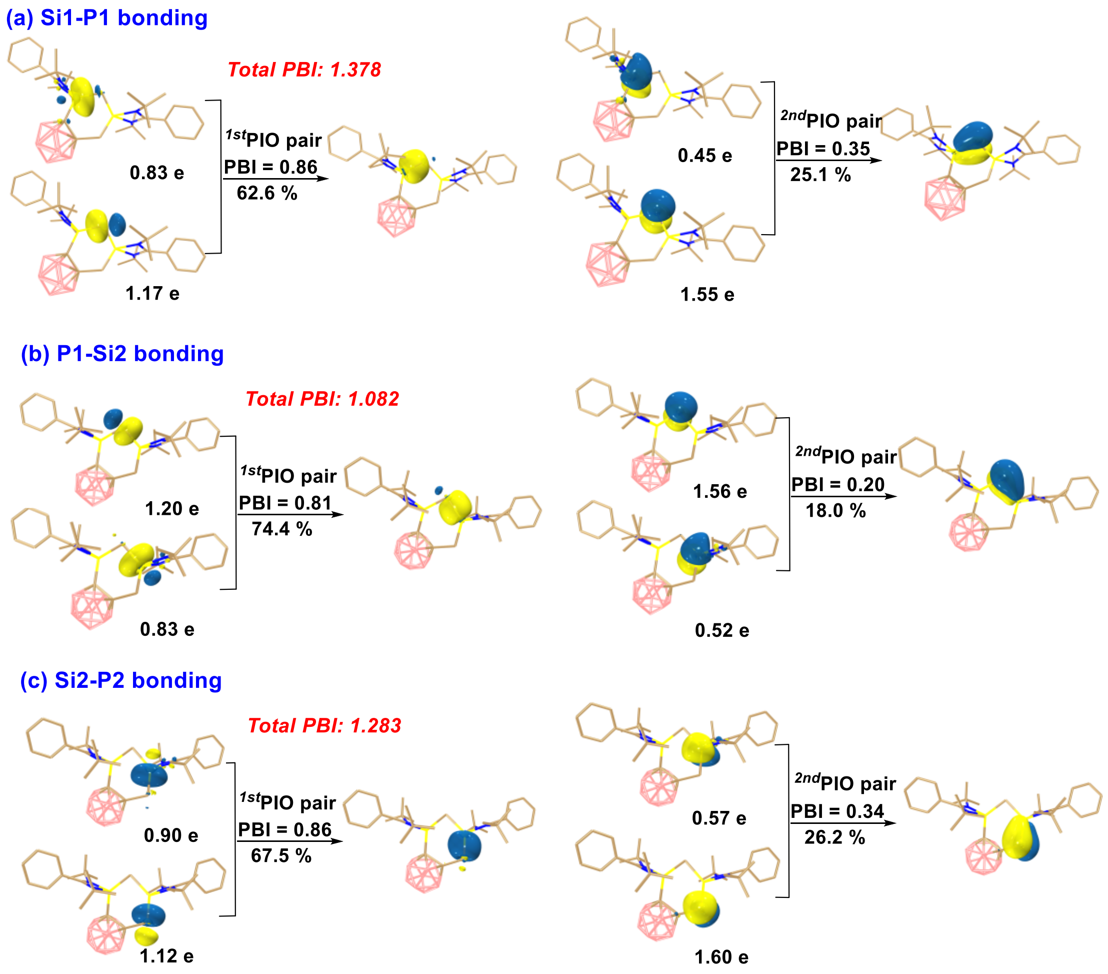

Figure S23. PIO analysis on the bonding modes between Si and P atoms in compound **4**. Hydrogen atoms in 3D structures are omitted for clarity. The PIO analysis is performed by cutting the Si-P bonds. The isosurface 0.050 au is plotted.

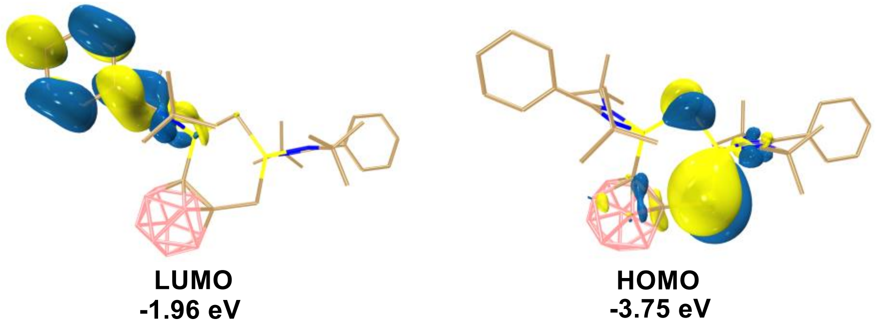

Figure S24. HOMO and LUMO orbitals of the compound **4**. Hydrogen atoms in 3D structures are omitted for clarity.

B4. Comparison of 2,4´and 4

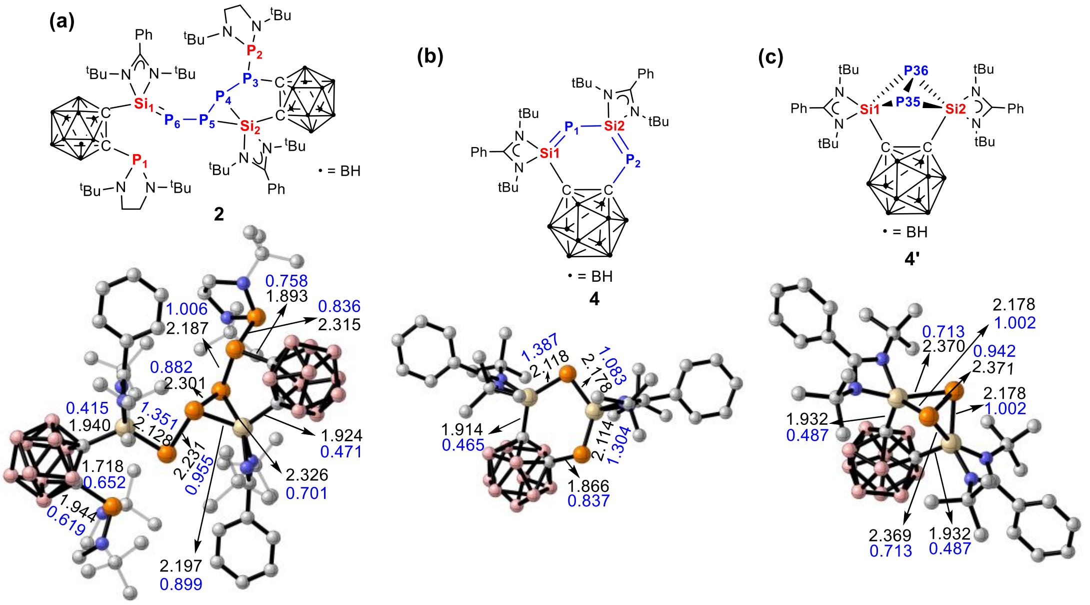

Figure S25. Structural parameters of compounds **2** (a), **4** (b), and **4´** (c) at TPSS-D3BJ / Def2-SVP ~ ma-TZVP level. Bond length (black) are in Å; Wiberg bond index (blue). Hydrogen atoms in 3D structures are omitted for clarity.

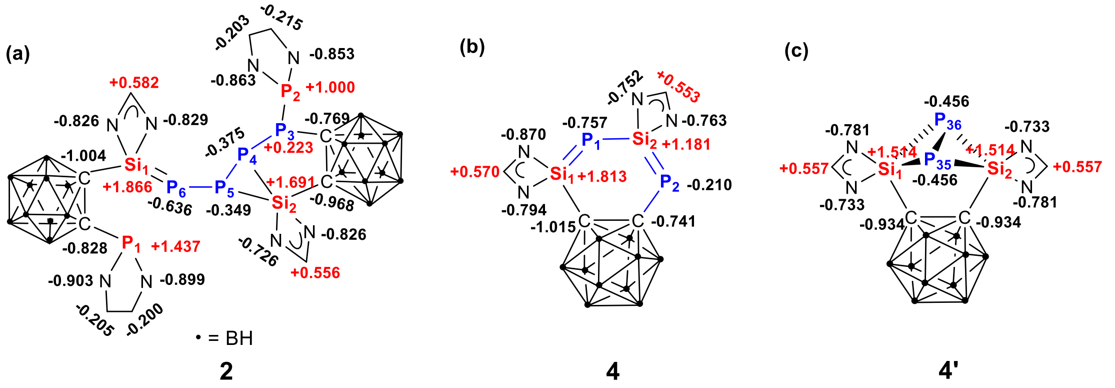

Figure S26. The natural population analysis of **2** (a), **4** (b), **4’** (c). Substituents in structures are omitted for clarity.

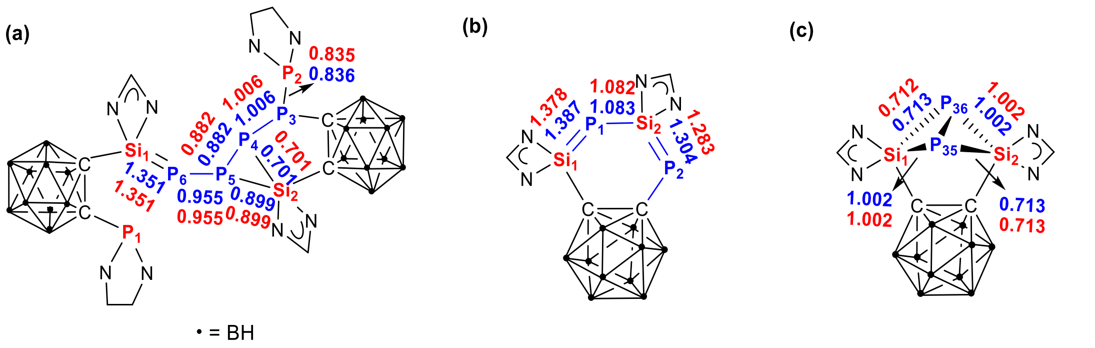

2 **4** **4'**

Figure S27. Comparison of PIO with NBO results of **2** (a), **4** (b), **4'** (c). The PBI (red) quantifies the strength of the interaction. Wiberg bond index is given in blue. Substituents in structures are omitted for clarity.

Cartesian Coordinates

2

TPSS-D3BJ/Def2-SVP~ma-TZVP

E = -5685.958376 a.u.

P	0.57423400	0.11027400	-0.75373500
P	3.70664500	-0.01811800	-0.19018700
Si	-1.73199600	-1.79543200	-0.57504100
P	-1.55704400	0.27525800	-0.11681500
P	1.97643200	0.56879600	1.01201700
Si	1.20709700	2.19396400	-0.46378400
P	5.44391200	-0.08750000	1.33826200
P	-4.75782600	-0.23176900	0.82995200
N	-0.06240000	3.27396200	-1.51190800
N	0.33411100	3.36957300	0.62602400
N	-0.42034600	-2.94665600	0.01834900
N	-0.88545600	-2.51006100	-2.05478800
N	-6.26033100	0.33676500	0.31016000
N	6.21930400	-1.40279600	0.55195900
C	-0.01195300	-3.14820800	-1.25083500
N	-5.26228200	-0.37500500	2.46268300
C	1.20608700	-3.88382000	-1.67166300
C	-3.40962200	-2.75884400	-0.43419600
C	-4.82664600	-2.09375600	0.27468900
C	4.13511100	1.62356200	-1.03052700
C	2.89034400	2.72008100	-1.23298600
C	-0.49279000	3.79857500	-0.37019400
N	4.77211800	-1.07196900	2.57245500
C	0.22212800	-3.31152500	1.31150600
C	1.18365100	-5.28498100	-1.79400300
H	0.25236800	-5.83238500	-1.61739000
C	-0.75611600	3.25860200	-2.83516000
C	-1.71425000	4.61642300	-0.16299300
C	-1.75064400	5.97965600	-0.50236200
H	-0.86418700	6.45656200	-0.93079500
C	5.44853100	-2.61055100	0.88982400
H	6.02559200	-3.52694000	0.69110400
H	4.54164600	-2.63412300	0.25595100
C	2.35583300	-5.96796300	-2.14740500
H	2.33838900	-7.05802600	-2.24670500
C	-0.85089100	-2.29843900	-3.53210500
B	3.48605500	4.31789000	-1.17672700
H	2.77407300	5.15606600	-0.70296200
C	-4.39737300	0.23035500	3.53073700
C	5.03213900	-2.49861000	2.36494100
H	4.13936900	-3.11629000	2.56339000
H	5.84368500	-2.85601500	3.03039500
C	0.32878400	3.69553700	2.08547200
C	7.70336700	-1.51133700	0.42373100
C	-5.19320300	1.59907200	-1.51236300
H	-4.54321400	2.14363500	-0.81120700
H	-5.38183300	2.24624800	-2.38477300

H	-4.64239800	0.70759300	-1.85317900
B	-4.74114300	-2.32982100	-1.41477700
H	-4.64839000	-1.37229000	-2.11829500
C	1.79048400	3.77490800	2.55906800
H	2.31008700	2.82450600	2.37658200
H	1.81291800	3.98387800	3.64199700
H	2.32811700	4.57908000	2.03089100
C	-0.11957300	2.12691700	-3.65877200
H	0.96344300	2.26486900	-3.77750600
H	-0.58159400	2.09396900	-4.65928500
H	-0.29527600	1.16192200	-3.15973400
C	-6.52706900	1.22184300	-0.85354700
B	3.43560500	1.81811400	-2.58680600
H	2.74441000	0.93604000	-2.99383000
C	4.42746900	-0.56546500	3.92550400
C	2.39794700	-3.16863000	-1.89816300
H	2.41843600	-2.08168800	-1.76378800
B	5.90071400	3.28904500	-2.37908400
H	7.01325500	3.47309300	-2.78918600
B	5.24623500	4.16728600	-0.95555200
H	5.87007900	4.98104800	-0.33380800
C	-7.33809800	-0.08387400	1.21324000
H	-7.74711600	-1.06992100	0.93042500
H	-8.16664800	0.64161900	1.18620500
B	-3.91764800	-3.26693500	1.11920600
H	-3.28431300	-2.92918200	2.06781200
B	3.06979000	3.54869400	-2.71514800
H	2.09024800	3.88236200	-3.29670600
B	4.15056300	3.06111400	-0.11077900
H	3.92754000	2.96423600	1.05188300
C	-2.91112400	6.72725500	-0.25984700
H	-2.93439300	7.79088400	-0.51782700
C	-3.99850100	4.75484000	0.65121100
H	-4.87247600	4.27488400	1.10139100
C	-2.84357500	4.00304100	0.41281900
H	-2.80363600	2.93524600	0.64980700
C	-6.71164100	-0.13303000	2.61492800
H	-6.87376300	0.83838000	3.12076600
H	-7.18522600	-0.91914600	3.22908000
B	-3.85902200	-3.84777900	-1.67769800
H	-3.14588300	-3.93581600	-2.62343700
B	-4.77585600	-4.81066000	0.86813900
H	-4.74663900	-5.65968400	1.71440100
C	-2.25965800	2.94118900	-2.70524700
H	-2.40278200	2.04436800	-2.07953100
H	-2.66570100	2.73525900	-3.71077600
H	-2.83280400	3.77764800	-2.27848800
B	5.18879800	1.65285800	-2.37451700
H	5.69549100	0.61589300	-2.69094900
B	-3.33792100	-4.43286100	-0.10019000
H	-2.26175700	-4.92328700	0.02600400
B	-4.74896500	-5.16347400	-0.88790600
H	-4.69025200	-6.28142500	-1.31867500
B	5.63146200	2.43867500	-0.84994700
H	6.44669400	1.96429200	-0.12673300
B	-5.69402100	-3.30630600	1.09717700
H	-6.26766900	-2.98994300	2.09529600
C	-0.41462600	2.57448800	2.83911100
H	-1.45447500	2.49105000	2.48871200
H	-0.42250200	2.78504500	3.92314600
H	0.07963900	1.60320800	2.67212900
C	3.56439800	-3.86002600	-2.24376700
H	4.48991700	-3.29766700	-2.40536400
C	-0.57354200	4.61673500	-3.54335500
H	-1.11051000	5.41345700	-3.00354000
H	-0.99155500	4.55811900	-4.56342800
H	0.48974100	4.89239800	-3.61616200
C	-4.98037100	-0.14182900	4.90758900
H	-5.95693400	0.33551400	5.09414400
H	-4.28919400	0.19151500	5.70050600
H	-5.10231500	-1.23552400	4.98565600
C	5.53089400	-0.94242300	4.93926600
H	5.62946300	-2.03633100	5.04845500
H	5.29566700	-0.52810200	5.93503700
H	6.50404900	-0.53495300	4.61302100
B	-5.62501500	-3.85139200	-1.73123400
H	-6.21433800	-3.99846400	-2.76502600
B	-6.20643900	-2.73085300	-0.49806400
H	-7.15303900	-2.02669800	-0.64222800
C	1.75408200	-3.20369200	1.25343100
H	2.21578600	-4.01317300	0.66902800
H	2.12944400	-3.27275900	2.28608000

H	2.06607300	-2.22803400	0.84576900
C	3.07174100	-1.16032800	4.36023400
H	2.29202900	-0.87478100	3.63469300
H	2.78878400	-0.78285300	5.35884800
H	3.11532000	-2.26199100	4.41949800
C	-2.06752600	-1.41222700	-3.85364400
H	-3.00708300	-1.92899300	-3.60641000
H	-2.07738600	-1.16813500	-4.92844300
H	-2.02338600	-0.47298800	-3.27637700
C	4.29831400	0.96622600	3.86750300
H	5.25948000	1.44611700	3.61906500
H	3.95621200	1.34864400	4.84423400
H	3.55569000	1.25703800	3.10402100
C	-0.31689100	5.06302600	2.39190100
H	0.10627500	5.85456700	1.75111400
H	-0.08436000	5.31417900	3.44061300
H	-1.41036500	5.06403000	2.28242900
C	-4.03453000	6.11792100	0.31775400
H	-4.93858300	6.70517600	0.50901600
C	-0.94982200	-3.64515200	-4.27630800
H	-0.08143000	-4.28489300	-4.04906100
H	-0.95708200	-3.45606500	-5.36350600
H	-1.86971900	-4.18868700	-4.01096200
C	-4.33113800	1.76439300	3.36368900
H	-3.86962700	2.00886600	2.39213600
H	-3.72337400	2.22732200	4.16004400
H	-5.33664800	2.21977100	3.39566100
C	-0.17470300	-4.74335500	1.72309900
H	-1.25636400	-4.82471300	1.90710700
H	0.35802200	-5.01285900	2.65143200
H	0.10645800	-5.46984600	0.94232600
C	-7.43492200	0.51390700	-1.88023200
H	-6.92818300	-0.36395300	-2.31037300
H	-7.69492600	1.20612400	-2.70035600
H	-8.37504900	0.17450700	-1.41155100
C	3.54550300	-5.25793500	-2.37026800
H	4.45970700	-5.79608500	-2.64176900
C	-2.98687100	-0.36062100	3.42562800
H	-3.01394100	-1.44194200	3.63173700
H	-2.31787100	0.12006500	4.15996600
H	-2.55367300	-0.19903000	2.42143800
B	4.53728600	2.90166800	-3.46629600
H	4.63890600	2.81299600	-4.65791500
B	-6.20751100	-4.47037600	-0.15210700
H	-7.23951500	-5.07144000	-0.03956400
C	0.44083700	-1.57663200	-3.97481200
H	0.66993700	-0.73068000	-3.30847600
H	0.30824800	-1.19673400	-5.00185000
H	1.30343000	-2.26002500	-3.97637700
C	8.28195000	-0.12368000	0.10839800
H	7.83777800	0.28264200	-0.81397500
H	9.37374300	-0.20188900	-0.02985200
H	8.08584000	0.58752600	0.92879200
B	4.57817700	4.46348200	-2.58614700
H	4.70978500	5.51150300	-3.15521800
C	-7.21294600	2.51621900	-0.35963500
H	-8.21832400	2.32170400	0.05136000
H	-7.33082600	3.22582400	-1.19625900
H	-6.60265800	2.99545100	0.42384900
C	8.01139800	-2.44755100	-0.76285800
H	7.63478800	-3.47062800	-0.58941100
H	9.10166500	-2.51720400	-0.92033400
H	7.54326700	-2.05522000	-1.68137400
C	-0.29515500	-2.30415600	2.34901700
H	0.04420400	-1.28457200	2.10571400
H	0.07893500	-2.57919300	3.34902200
H	-1.39388200	-2.30880300	2.37591600
C	8.34657700	-2.05418700	1.71956700
H	8.11758600	-1.38659700	2.56875300
H	9.44406700	-2.11781500	1.61459700
H	7.97505400	-3.06525100	1.96147600

4

TPSS-D3BJ/Def2-SVP~ma-TZVP

E = -2982.177129 a.u.

Si	-1.34459000	-0.12275100	0.16731100
P	0.37289100	-1.17688800	0.82067300
Si	1.78698400	0.16200400	-0.15392000
P	1.41876000	1.79728300	-1.49086500
N	-2.79380100	-0.46811100	1.23822900
N	-2.63131100	-0.94825100	-0.86760500

N	3.28373900	0.21968300	0.98910000	H	-3.18812200	-3.27952700	-3.31891600
N	3.21216500	-0.73275700	-0.94859300	H	-4.34374500	-2.96274200	-2.00128700
C	-4.84403700	-1.54548300	0.21128100	C	5.83350700	-2.05433000	0.78650400
C	-0.15609900	2.58100600	-0.86715900	H	5.06266700	-2.75648300	1.11810300
C	-1.50489400	1.73761900	-0.25278100	C	7.79893500	-0.25792800	-0.10126000
C	5.45593500	-0.82040500	0.22470700	H	8.56391300	0.44471400	-0.44708100
C	-3.45017300	-1.02561300	0.19702000	C	2.42634300	1.53028200	2.82098100
C	-5.89136700	-0.63632900	-0.03524700	H	2.44326000	2.43829800	2.19615900
H	-5.65745400	0.41481800	-0.22826800	H	2.55255700	1.82076300	3.87745200
C	-3.04822500	-0.59282000	2.70253500	H	1.44275900	1.04533300	2.70356100
C	4.01895800	-0.45792200	0.09719000	C	3.52497800	-1.23001900	-2.30970800
C	-2.77511800	-1.43246400	-2.26411600	C	-3.73203000	-0.52564400	-3.06167900
C	6.44455300	0.07886400	-0.21798200	H	-4.73804200	-0.52692900	-2.60965100
H	6.14686700	1.04253900	-0.64209900	H	-3.81911000	-0.90192000	-4.09552800
C	-4.44057400	-0.04687000	3.07043300	H	-3.36160800	0.50917300	-3.09577100
H	-4.55873800	0.98723500	2.70690800	C	8.17375700	-1.48800500	0.45972700
H	-4.54969700	-0.04536300	4.16846800	H	9.23325000	-1.74837300	0.55125300
H	-5.24876900	-0.66546500	2.64935400	B	-1.54589600	2.29621200	-1.86292200
C	-7.21938900	-1.08073000	-0.01868300	H	-1.43100300	1.52071000	-2.75670400
H	-8.02935000	-0.36975300	-0.20983800	B	-0.61538300	2.78340700	0.77620200
C	-5.13535100	-2.89747300	0.47033700	H	0.05765100	2.28778500	1.62220100
H	-4.32153300	-3.60656800	0.64601400	B	-2.39394000	2.73538300	0.82085500
C	-7.50913200	-2.42736700	0.24569900	H	-2.93742100	2.22343200	1.74756700
H	-8.54823800	-2.77166200	0.26102100	C	7.18974100	-2.38382300	0.90384800
C	-1.35460500	-1.40074500	-2.85405800	H	7.47848000	-3.34517000	1.34091200
H	-0.89501300	-0.40346300	-2.74608500	B	-1.55767000	4.29514200	0.85198500
H	-1.38572600	-1.65349500	-3.92693400	H	-1.53647800	4.96174000	1.84920300
H	-0.71073600	-2.12620300	-2.33249900	B	-3.02265000	4.07501000	-0.15360900
C	-1.95824600	0.21136700	3.43673200	H	-4.07206800	4.58812200	0.12276400
H	-0.95502300	-0.14109600	3.14393400	B	-0.14006400	4.17232100	-0.21129500
H	-2.08130300	0.07099100	4.52358700	H	0.93278900	4.66164600	-0.01902700
H	-2.03036100	1.28578400	3.21346500	B	-2.97375000	2.42621900	-0.81608100
C	3.54575800	0.56121700	2.40460100	H	-3.89307100	1.68513500	-1.00695500
C	-6.46634500	-3.33340600	0.48899900	B	-0.71419300	3.86022000	-1.86154600
H	-6.68807700	-4.38596800	0.69206400	H	-0.03653200	4.13226400	-2.80800000
C	-2.91269000	-2.07757800	3.10375900	C	2.17955900	-1.66085300	-2.91881800
H	-3.70044500	-2.69123900	2.63828200	H	1.73021600	-2.47221800	-2.32186900
H	-3.00578100	-2.17960800	4.19865500	H	2.33075800	-2.01603600	-3.95226600
H	-1.92551800	-2.46382400	2.79674800	H	1.48264200	-0.80664100	-2.93080700
C	-3.28458200	-2.88828900	-2.29176500	B	-1.62146400	4.96480700	-0.80211700
H	-2.67998700	-3.52355400	-1.62183200	H	-1.64179500	6.14471000	-1.01937900

B	-2.49446500	3.79206400	-1.83843900
H	-3.13902400	4.10565000	-2.80055200
C	4.13956500	-0.08699200	-3.14413700
H	3.45924600	0.78184900	-3.16027700
H	4.31115700	-0.42232600	-4.18199600
H	5.10797700	0.22792000	-2.71994000
C	4.91220400	1.25124700	2.58339000
H	5.75223600	0.56268900	2.40233000
H	4.99483500	1.62212400	3.61965300
H	5.00343300	2.11142100	1.89857600
C	4.47452500	-2.44341100	-2.27259100
H	5.49485800	-2.16843100	-1.96414800
H	4.53337300	-2.87801300	-3.28537700
H	4.09223000	-3.21781700	-1.58550000
C	3.46507400	-0.72131200	3.25994800
H	2.49208300	-1.21824100	3.10393800
H	3.56904300	-0.47271900	4.33070900
H	4.27182200	-1.42408500	2.99179600

4´

TPSS-D3BJ/Def2-SVP~ma-TZVP

E = -2982.187937 a.u.

Si	-1.45993700	0.04023700	-0.42564400
N	3.07060900	-1.10052700	-0.18772800
C	0.82526400	0.00468300	1.39868900
B	-0.00563400	1.42855700	1.85847800
H	-0.00102600	2.34440200	1.11001900
Si	1.46004600	-0.04010500	-0.42513900
N	2.97144400	1.03470900	-0.58114900
C	-0.82571100	-0.00441800	1.39839500
B	0.00507700	-1.42829200	1.85834200
H	0.00073200	-2.34406800	1.10980400
N	-2.97118800	-1.03475800	-0.58146200
C	-3.81181600	0.01243000	-0.39531700
B	-1.43802600	-0.88894000	2.72663000
H	-2.45848700	-1.49660300	2.57599100
N	-3.07068000	1.10055400	-0.18858700
B	1.43718000	0.88915300	2.72718100
H	2.45770700	1.49675800	2.57677100
B	-1.44195800	0.87998200	2.73266000
H	-2.46580700	1.47955500	2.59276200
B	1.44110400	-0.87979500	2.73308800
H	2.46494000	-1.47945700	2.59345500
B	0.00271100	-1.44668400	3.62361300
H	0.00649700	-2.49373400	4.20905300
B	-0.89340300	-0.00275700	4.17652500
H	-1.54759700	-0.00477300	5.18277300
B	-0.00387100	1.44678100	3.62373900
H	-0.00795000	2.49381400	4.20921100
C	-3.27945200	-2.46916100	-0.86353600
B	0.89209100	0.00281800	4.17681800
H	1.54596400	0.00460200	5.18327500
C	-3.47210500	2.52515500	-0.35692000
C	3.81191300	-0.01256100	-0.39479200
C	3.47189400	-2.52518800	-0.35612900
C	3.28002900	2.46918200	-0.86257800
P	-0.11668800	1.17980200	-1.70576700
P	0.11729000	-1.17959000	-1.70586800
C	-5.29718300	-0.04390600	-0.39419100
C	-6.02209900	-0.03298300	-1.59978400
C	-5.98007500	-0.10913000	0.83517900
C	-7.42230700	-0.08325800	-1.57325300
H	-5.48966300	0.00986800	-2.55467100
C	-7.37770800	-0.16986700	0.85442400
H	-5.40845800	-0.11525800	1.76829000
C	-8.10138600	-0.15491900	-0.34825100
H	-7.98320200	-0.07076900	-2.51333500
H	-7.90482200	-0.22615700	1.81223100
H	-9.19518100	-0.19891800	-0.33010200
C	5.29728600	0.04362300	-0.39411900
C	6.02168500	0.03336700	-1.60004200
C	5.98073100	0.10813000	0.83497300
C	7.42189900	0.08368600	-1.57410900
H	5.48882100	-0.00907000	-2.55470600
C	7.37837100	0.16894400	0.85363300
H	5.40954800	0.11368200	1.76835200
C	8.10151700	0.15473700	-0.34936700
H	7.98237800	0.07170700	-2.51444400
H	7.90589600	0.22472200	1.81124300
H	9.19531600	0.19880800	-0.33167800
C	2.19449100	3.35242500	-0.22088000

H	1.19475800	3.09213700	-0.59779600
H	2.19938700	3.24104000	0.87443500
H	2.39919800	4.40763100	-0.46905100
C	4.63148300	2.92628500	-0.27571200
H	4.69030200	4.02288200	-0.38208300
H	4.69971500	2.68417400	0.79785600
H	5.49928900	2.49319600	-0.79297200
C	3.27222600	2.65115400	-2.39504200
H	4.06185300	2.03742500	-2.86184700
H	2.29537300	2.34641900	-2.80814800
H	3.45296900	3.70817600	-2.65779600
C	3.72976500	-2.78255700	-1.85736100
H	3.96142400	-3.84866400	-2.02689600
H	2.83447400	-2.51734300	-2.44580500
H	4.58433200	-2.18632400	-2.21825800
C	2.30334600	-3.40995100	0.10818400
H	2.11465700	-3.28469000	1.18441500
H	1.38159100	-3.16387100	-0.44318900
H	2.55292300	-4.46665700	-0.08545000
C	4.71538100	-2.89183600	0.47930800
H	5.63720500	-2.43740700	0.08681500
H	4.58342200	-2.58057100	1.52923100
H	4.84437600	-3.98788100	0.45853300
C	-2.30375300	3.41001200	0.10768300
H	-2.11531600	3.28475500	1.18395600
H	-1.38184100	3.16402900	-0.44347300
H	-2.55339200	4.46669400	-0.08599200
C	-3.72968100	2.78257600	-1.85820600
H	-2.83421500	2.51753400	-2.44646100
H	-4.58405700	2.18620500	-2.21932500
H	-3.96147400	3.84865200	-2.02774500
C	-4.71583000	2.89162700	0.47824400
H	-4.58406900	2.58040000	1.52820300
H	-4.84497700	3.98765300	0.45741500
H	-5.63750200	2.43704600	0.08556400
C	-3.27071200	-2.65053200	-2.39606600
H	-2.29355100	-2.34578700	-2.80843900
H	-3.45147900	-3.70741000	-2.65936200
H	-4.05993300	-2.03643800	-2.86311200
C	-2.19420800	-3.35252900	-0.22151600
H	-1.19427700	-3.09199000	-0.59774300

H	-2.19977500	-3.24156600	0.87383300
H	-2.39863500	-4.40766600	-0.47022900
C	-4.63121900	-2.92661700	-0.27768600
H	-4.70014500	-2.68493900	0.79593200
H	-5.49872900	-2.49337800	-0.79530100
H	-4.68990300	-4.02317400	-0.38454600
C	4.13956500	-0.08699200	-3.14413700
H	3.45924600	0.78184900	-3.16027700
H	4.31115700	-0.42232600	-4.18199600
H	5.10797700	0.22792000	-2.71994000
C	4.91220400	1.25124700	2.58339000
H	5.75223600	0.56268900	2.40233000
H	4.99483500	1.62212400	3.61965300
H	5.00343300	2.11142100	1.89857600
C	4.47452500	-2.44341100	-2.27259100
H	5.49485800	-2.16843100	-1.96414800
H	4.53337300	-2.87801300	-3.28537700
H	4.09223000	-3.21781700	-1.58550000
C	3.46507400	-0.72131200	3.25994800
H	2.49208300	-1.21824100	3.10393800
H	3.56904300	-0.47271900	4.33070900
H	4.27182200	-1.42408500	2.99179600

References

1. King, R. B.; and Sundaram, P. M. Bis(dialkylamino)phosphines. *J. Org. Chem.* **1984**, *49*, 1784-1789.
2. Sen, S. S.; Roesky, H. W.; Stern, D.; Henn, J.; and Stalke, D. High Yield Access to Silylene RSiCl (R) PhC(NtBu)2) and Its Reactivity toward Alkyne: Synthesis of Stable Disilacyclobutene. *J. Am. Chem. Soc*., **2010**, *132*, 1123-1126.
3. Sheldrick, G. M. *SHELX-97 Program for Crystal Structure Determination*, Universität Göttingen, Germany (1997).
4. Frisch, M. J.; Trucks, G. W.; Schlegel, H. B.; Scuseria, G. E.; Robb, M. A.; Cheeseman, J. R.; Scalmani, G.; Barone, V.; Mennucci, B.; Petersson, G. A.; Nakatsuji, H.; Caricato, M.; Li, X.; Hratchian, H. P.; Izmaylov, A. F.; Bloino, J.; Zheng, G.; Sonnenberg, J. L.; Hada, M.; Ehara, M.; Toyota, K.; Fukuda, R.; Hasegawa, J.; Ishida, M.; Nakajima, T.; Honda, Y.; Kitao, O.; Nakai, H.; Vreven, T.; Montgomery, J. A., Jr.; Peralta, J. E.; Ogliaro, F.; Bearpark, M.; Heyd, J. J.; Brothers, E.; Kudin, K. N.; Staroverov, V. N.; Kobayashi, R.; Normand, J.; Raghavachari, K.; Rendell, A.; Burant, J. C.; Iyengar, S. S.; Tomasi, J.; Cossi, M.; Rega, N.; Millam, J. M.; Klene, M.; Knox, J. E.; Cross, J. B.; Bakken, V.; Adamo, C.; Jaramillo, J.; Gomperts, R.; Stratmann, R. E.; Yazyev, O.; Austin, A. J.; Cammi, R.; Pomelli, C.; Ochterski, J. W.; Martin, R. L.; Morokuma, K.; Zakrzewski, V. G.; Voth, G. A.; Salvador, P.; Dannenberg, J. J.; Dapprich, S.; Daniels, A. D.; Farkas, O.; Foresman, J. B.; Ortiz, J. V.; Cioslowski, J.; Fox, D. J., Gaussian 16, Revision A.03; Gaussian, Inc., Wallingford CT, **2016**.
5. Tao, J. M., Perdew, J. P., Staroverov, V. N., Scuseria. G. E., Climbing the density functional ladder: Nonempirical meta-generalized gradient approximation designed for molecules and solids. *Phys. Rev. Lett.* **2003**, *91*, 146401.
6. Weigend F., Ahlrichs. R., Balanced basis sets of split valence, triple zeta valence and quadruple zeta valence quality for H to Rn: Design and assessment of accuracy. *Phys. Chem. Chem. Phys.* **2005**, *7*, 3297-305.
7.Zheng, J.; Xu, X.; Truhlar, D. G. Minimally augmented Karlsruhe basis sets. *Theor. Chem. Acc.* **2010**, *128*, 295-305.
8. Papajak, E.; Zheng, J.; Xu, X.; Leverentz, H. R.; Truhlar, D. G. Perspectives on Basis Sets Beautiful: Seasonal Plantings of Diffuse Basis Functions. *J. Chem. Theory. Comput.* **2011**, *7*, 3027-3034.
9. Wilson, P. J.; Bradley, T. J.; Tozer, D. J., Hybrid exchange-correlation functional determined from thermochemical data and ab initio potentials. *J. Chem. Phys.* **2001**, *115*, 9233-9242
10. Weigend, F.; Ahlrichs, R., Balanced basis sets of split valence, triple zeta valence and quadruple zeta valence quality for H to Rn: Design and assessment of accuracy. *Phys. Chem. Chem. Phys.* **2005**, *7*, 3297-3305.
11. Jameson, C. J.; De Dios, A.; Keith Jameson, A., Absolute shielding scale for ^{31}P from gas-phase NMR studies. *Chem. Phys. Lett.* **1990**, *167*, 575-582.
12. C. Y. Legault, CYLview, 1.0b ed.; Université de Sherbrooke: Sherbrooke, Québec, Canada, **2009**; http://www.cylview.org.
13. Humphrey, W.; Dalke, A.; Schulten, K. VMD: Visual molecular dynamics. *J. Mol. Graphics* **1996**, *14*, 33-38.
14. Lu, T.; Chen, F., Multiwfn: a multifunctional wavefunction analyzer. *J. Comput. Chem.* **2012**, *33*, 580-592.
15. E. D. Glendening, J. K. Badenhoop, A. E. Reed, J. E. Carpenter, J. A. Bohmann, C. M. Morales, C. R. Landis, F. Weinhold, NBO 7.0; Theoretical Chemistry Institute, University of Wisconsin: Madison, WI, **2013**. http://nbo7.chem.wisc.edu/.
